# Pharmacological investigation of new niclosamide-based isatin hybrids as antiproliferative, antioxidant, and apoptosis inducers

**DOI:** 10.1038/s41598-024-69250-5

**Published:** 2024-08-27

**Authors:** Mervat M. Omran, Mona M. Kamal, Yousry A. Ammar, Moustafa S. Abusaif, Magda M. F. Ismail, Heba H. Mansour

**Affiliations:** 1 Pharmacology Unit, Cancer Biology Department, National Cancer Institute, Cairo University, Cairo, Egypt; 2 Pharmacology and Toxicology Department, Faculty of Pharmacy, Al-Azhar University, Cairo, Egypt; 3 Department of Chemistry, Faculty of Science (Boys), Al-Azhar University, Nasr City, Cairo, 11884 Egypt; 4 Department of Medicinal Pharmaceutical Chemistry and Drug Design, Faculty of Pharmacy (Girls), Al-Azhar University, Nasr City, Cairo, 11754 Egypt; 5 Health Radiation Research Department, National Centre for Radiation Research and Technology, Egyptian Atomic Energy Authority, P.O. Box 29, Nasr City Cairo, Egypt

**Keywords:** Niclosamide analogue synthesis, Cytotoxicity, Apoptosis, Cell cycle, Antioxidant, Molecular docking, Biochemistry, Cancer

## Abstract

A group of Niclosamide-linked isatin hybrids (Xo, X1, and X2) was created and examined using IR, ^1^HNMR, ^13^C NMR, and mass spectrometry. These hybrids' cytotoxicity, antioxidant, cell cycle analysis, and apoptosis-inducing capabilities were identified. Using the SRB assay, their cytotoxicity against the human HCT-116, MCF-7, and HEPG-2 cancer cell lines, as well as VERO (African Green Monkey Kidney), was evaluated. Compound X1 was the most effective compound. In HCT-116 cells, compound X1 produced cell cycle arrest in the G1 phase, promoted cell death, and induced apoptosis through mitochondrial membrane potential breakdown in comparison to niclosamide and the control. Niclosamide and compound X1 reduced reactive oxygen species generation and modulated the gene expression of BAX, Bcl-2, Bcl-xL, and PAR-4 in comparison to the control. Docking modeling indicated their probable binding modalities with the XIAP BIR2 domain, which selectively binds caspase-3/7, and highlighted their structural drivers of activity for further optimization investigations. Computational in silico modeling of the new hybrids revealed that they presented acceptable physicochemical values as well as drug-like characteristics, which may introduce them as drug-like candidates. The study proved that compound X1 might be a novel candidate for the development of anticancer agents as it presents antiproliferative activity mediated by apoptosis.

## Introduction

The programmed death of cells known as apoptosis is essential for eradicating abnormal cells that endanger growth, balance, and life itself^1^. Apoptosis can be dysregulated to cause ischemic heart disease (acute myocardial infarction), Parkinson's disease, Alzheimer's disease, and Huntington's disease, among other neurodegenerative diseases^2,3^. On the other hand, one characteristic linked to cancer is the capacity of malignant cells to evade apoptosis^4^. This is why several therapeutic approaches entail stimulating the caspase cascade to induce apoptosis in tumors^5,6^.

Through the technique of drug repurposing, creative uses for medications that are marketed for a variety of therapeutic aims have recently come to light. The danger of failure has been significantly mitigated because the safety profile of these medications has been thoroughly examined in the past, the creation of their formulation has been examined, and they have successfully completed the preclinical and clinical phases of research. An anthelmintic medication licensed by the FDA for the treatment of parasitic illnesses is Niclosamide. However, recent medication repurposing has highlighted that, over the past few years, increasing evidence has shown that Niclosamide could treat diseases beyond parasitic diseases, including the treatment of Parkinson's disease, diabetes, viral and microbial infections, as well as several malignancies^7–9^.

The basic mechanisms of niclosamide are believed to be the activation of ATPase activity or the uncoupling of mitochondrial oxidative phosphorylation^7^. Niclosamide has been shown to impact other signaling pathways, apart from Wnt/β-catenin, mTOR, JAK/STAT3, and NF-B. This naturally links niclosamide's therapeutic potential to numerous illnesses that involve these important signaling cascades^8^. Primary human glioblastoma cells' malignant potential can be dramatically reduced in vivo by niclosamide through the suppression of intracellular Wnt/β-catenin, NOTCH-, mTORC1, and NF-kB signaling cascades^8^. The ability of niclosamide to damage the mitochondria of tumor cells, inhibit many signal pathways that control the initiation and progression of cancer, induce cancer cell cycle arrest in the G1 phase, and induce growth inhibition and apoptosis in cancer cells is linked to its anticancer activity^7,8,10^.

Niclosamide not only blocked the activation of the STAT3/Bcl-2/BCL-xl pathway induced by ionizing radiation in both radiosensitive and radioresistant human lung cancer cells but also overcame acquired radioresistance in vitro and in vivo^11^. Previous studies reported that niclosamide inhibits TNF-α-induced NF-κB-dependent activity, increases reactive oxygen species (ROS) levels in AML cells, and enhances the sensitivity of lung cancer cells to ROS^12,13^.

Additionally, Schiff's bases with imine functional groups make hydrogen bonds with the active centers of each individual cell. Their contribution to the development of innovative drugs with strong anticancer properties and few side effects is significant^14,15^. It is also commonly recognized that sunitinib contains the bioactive isatin moiety, which stops the growth of tumor cells that produce medulloblastoma and induces apoptosis. It has been discovered that certain isatin-Schiff bases, isatin-based compounds, and isatin-5-sulfonamides display strong, selective, and antiproliferative non-peptide caspase-3 and -7 inhibitors^16–19^.

This work utilized molecular hybridization, a useful technology in drug optimization for cancer therapy, which allows the fusing of one or more bioactive chemicals (Niclosamide) or pharmacophoric subunits (isatin) into a single molecule^20^. Additionally, we employ Schiff's base linker (–CH=N–), a bioisostere to the linker found in sunitinib (–C=CH–), Supplementary S1. Medicinal chemists employ this technique to rationally transform lead compounds into safer, more cost-effective, clinically useful, and therapeutically appealing medications. Lastly, in order to probe for the additional binding affinity of our hybrids, sulfonyl piperidine or sulfonyl N-methyl piperazine will be added at p-5 of isatin's phenyl using extension and ring variation techniques. The current study aimed to synthesize novel hybrids of Niclosamide and isatin and assess their antiproliferative, antioxidant, and apoptotic mechanisms associated to oxidative stress.

## Results

### Chemical results

Supplementary materials S2 and S3 include the synthetic procedures used to produce the target compounds Xo, X1, and X2. Using two processes, a good yield (100%) of N-(4-amino-2-chlorophenyl)-5-chloro-2-hydroxybenzamide, which is used as an intermediate, was produced. To obtain the corresponding 5-chloro-2-hydroxybenzoyl chloride, 5-chloro-2-hydroxybenzoic acid and 2-chloro-4-aminoaniline were reacted with in the first step. This produced the desired compound, 5-chloro-N-(2-chloro-4-nitrophenyl)-2-hydroxybenzamide (Niclosamide). In the second stage, N-(4-amino-2-chlorophenyl)-5-chloro-2-hydroxybenzamide (Niclosamide amine) was prepared by reducing the nitro functionality of Niclosamide. Ultimately, condensation of Niclosamide amine 2 with different heterocyclic ketones namely, indoline-2,3-dione, (5-(piperidin-1-ylsulfonyl)indoline-2,3-dione, or 5-((4-methylpiperazin-1-yl)sulfonyl)indoline-2,3-dione) in acetic acid medium afforded the corresponding new Niclosamide-isatin hybrids, **X**_**o**_**, X**_**1**_ and** X**_**2**_ via Schiff’s base linker. All newly synthesized compounds' spectroscopic data, including elemental analysis, ^1^H NMR, ^13^C NMR, and IR, were consistent with the proposed structure. Compound **Xo**'s infrared spectrum showed bands at v 3220, 3182, 3159, 1708, and 1631 cm-1 that were associated with the OH, 2NH, C=O, and novel C=N groups, respectively. In addition to the aromatic protons, their ^1^H NMR spectra showed three exchangeable singlet signals at *δ* 7.06, 10.91, 11.02 ppm related to hydroxyl group and two NH functions. Additionally, compound **Xo**'s ^13^CNMR spectrum presented sharp signals due to C–OH, C=N, and two carbonyl groups at *δ* 156.15, 160.00, 163.65 and 163.83 ppm, respectively, as well as signals for aromatic carbons.

Additionally, very significant absorption bands at v 3456, 3438, and 3309 cm-1 attributed to the OH and 2NH groups, respectively, were revealed by infrared analysis of compound **X1**. Likewise bands corresponding to carbonyl, aliphatic piperidinyl, and C=N functionalities emerged at ν 2940, 2856, 1734, and 1616 cm-1, in that order. In addition, the ^1^H NMR spectrum showed the presence of aromatic protons and three exchangeable signals at δ 7.05, 11.36, and 11.47 ppm, which corresponded to two OH and two NH, respectively, as well as three novel signals caused by the piperidinyl protons at *δ* 1.32, 1.46, and 2.65 ppm. Compound X1's ^13^CNMR analysis revealed three signals at δ 23.41, 25.31, and 47.17 ppm that were attributed to the carbons of the piperidinyl group; two carbonyl groups that appeared at δ 163.09 and 164.27 ppm, respectively; and signals at δ 129.05 and 134.45 ppm that were related to (2C–Cl) functions and signal at δ 159.93 ppm due to the C=N group. X2's spectrum was almost identical to X1's; however, 1H NMR analysis showed a singlet at δ 2.02 ppm because of N-CH3 functionality, which is also present in 13C NMR at the aliphatic region (δ 44.1 ppm). Mass spectra confirmed the structures of Xo and X1 and their suggested fragmentation pattern according to Supplementary S4.

### Biochemical results

#### Antiproliferative screening

The antiproliferative profile of the synthesized compounds was evaluated in vitro against human colon carcinoma HCT116, HEPG-2, and MCF-7, as well as VERO (African Green Monkey Kidney) as a normal cell line to evaluate the safety of our hybrids. The present study showed that Niclosamide and the three Niclosamide-isatin hybrids showed promising cytotoxic activities on MCF-7, HepG-2, and HCT-116 cell lines.

**Figure 1:**
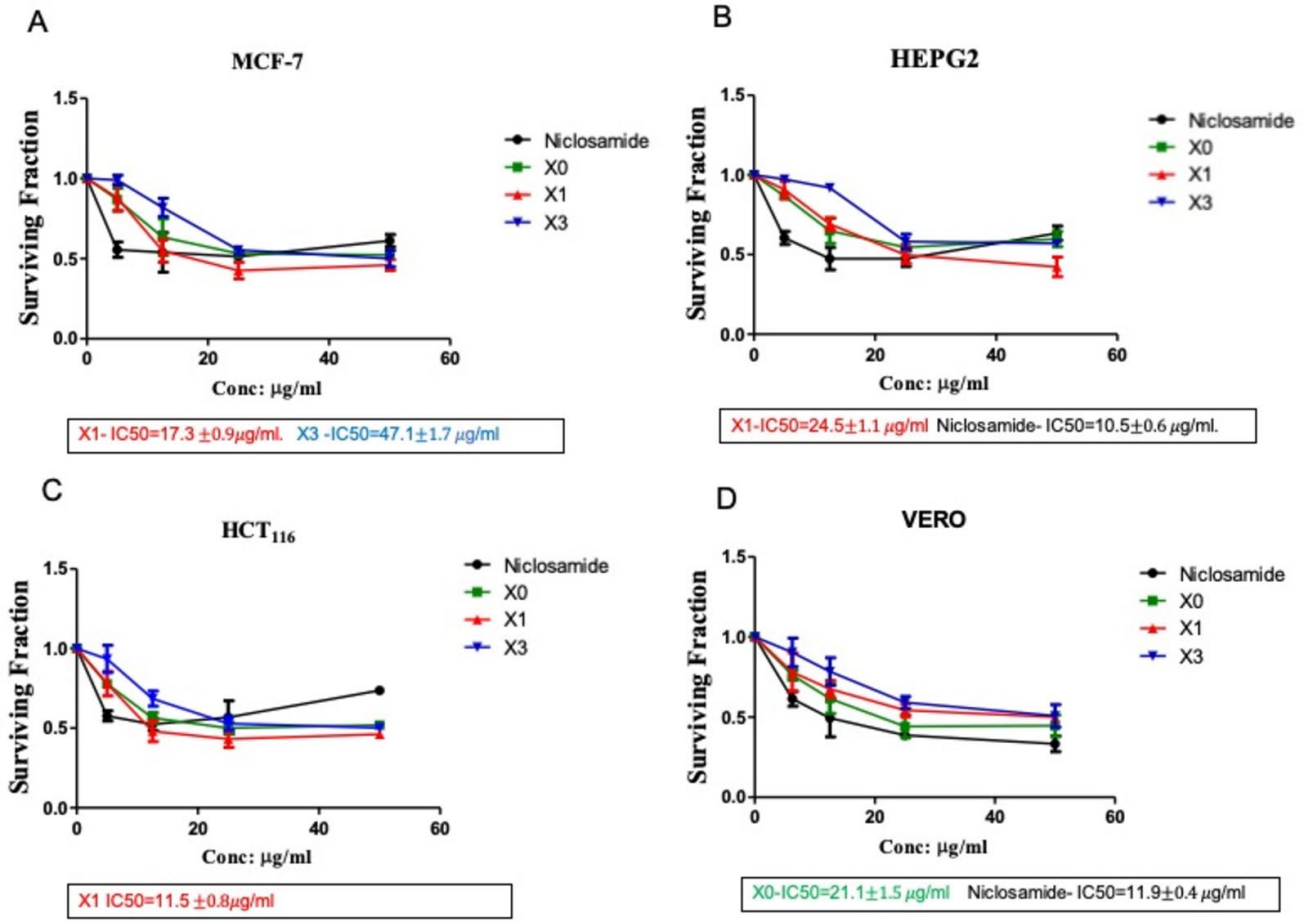
Cytotoxicity of Niclosamide as the reference standard and Niclosamide-isatin hybrids X0, X1, and X2 on the MCF-7 breast Cancer cell line (**A**), HEPG2 liver cancer cell line (**B**), the HCT-116 human colorectal carcinoma cell line (**C**), and the VERO African Green Monkey Kidney normal cell (**D**). Values are represented as means ± SD of three experiments (independent and triplicate).

Compounds X1 and X2 show anticancer activity on MCF-7 cell lines with IC_50_ equivalent to 30.16 and 80.03 µM, respectively (Fig. 1A); X1 exerts a better effect on the MCF-7 cell line. Niclosamide and X1 showed promising anticancer effects on HEPG-2 cell lines with IC_50_ of 32.09 and 42.72 µm, respectively (Fig. 1B), where Niclosamide exerts a better effect on the HEPG-2 cell line. Compound X1 shows a promising anticancer effect on the HCT-116 cell line with IC_50_ of 20.05 µM (Fig. 1). The selectivity of compounds against cancer cell lines was confirmed by testing their cytotoxicity on VERO (African Green Monkey Kidney) normal cells. Compounds X1 and X2 are found to be safe and selective, as no IC_50_ was detected on the normal cell line at 50 µg/ml, which corresponds to 87.19 and 84.96 µM, respectively. Niclosamide and Xo were the most toxic on the normal cell line, with IC_50_ of 36.37 and 49.50 µM, respectively (Fig. 1). To our delight, they were found to be inactive against this normal cell (IC_50_ > 84.96–87.19 μM), revealing that they possessed a large safety margin to selectively target cancerous cells other than normal cells as shown by their specified IC_50_ values. Compound X1 was highly selective for colon cancer cell lines. Due to this, **X1** was chosen for further investigations compared to Niclosamide.

#### Effect of niclosamide and its hybrid on glutathione content (GSH), total nitrate/nitrite (NOx), and lipid peroxidation (MDA)

**Figure 2:**
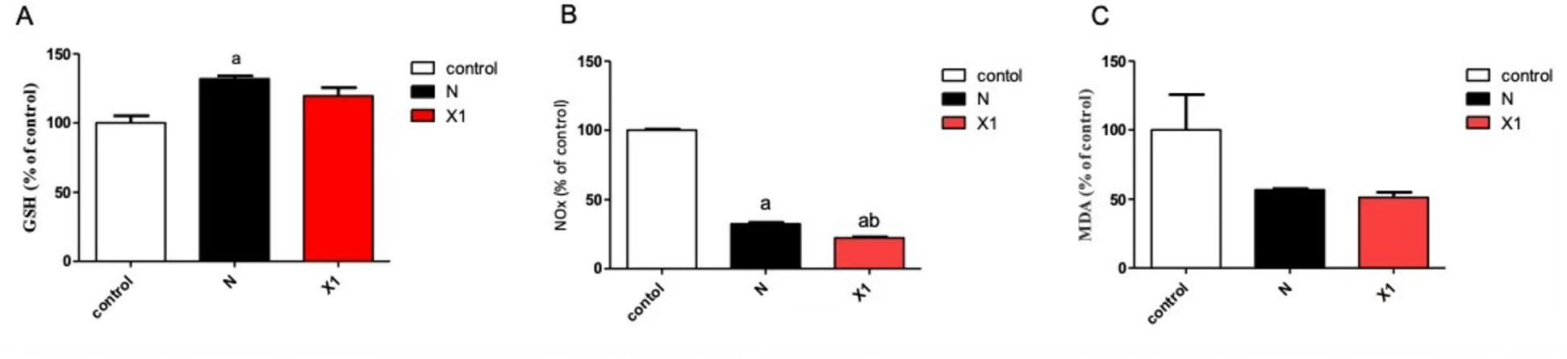
Effect of treatment with control, niclosamide, and compound X1 on oxidative stress and antioxidants in the HCT-116 cell line. GSH content (**A**), NOx (**B**) level, and MDA level (**C**). The data were expressed as the means ± SD of three independent experiments. a: significantly different from the control group; b: significantly different from niclosamide treated group at a P value < 0.05.

The tested niclosamide significantly increased the GSH level (31.9%) compared to untreated cells, accompanied by a significant reduction in the NOx (68.6%) and no significant decrease in MDA (44.2%) content in HCT-116 cells (Fig. 2) compared to untreated cells. Compound X1 shows no significant decrease in MDA (48.8%) level and a significant reduction in NOx level (77.7%) compared to both control and niclosamide, and that may indicate the more tolerated and less toxic effect of the compound X1. Reactive oxygen species (ROS) production after treatment with these compounds was found to be decreased.

#### mRNA expression of apoptotic genes

Interestingly, our investigation using real-time PCR analysis showed that Niclosamide (N) and our hit (X1) induced apoptotic effects via significant downregulation of anti-apoptotic Bcl-2 (P = 0.01, 0.005), an insignificant decrease of B-cell lymphoma extra-large (Bcl-xl, P = 0.5, 0.1), and significant upregulation by a two- and five-fold increase of apoptotic genes PAR-4 (P = 0.008, 0.007) and a four-fold and a threefold increase in BAX (P = 0.001, 0.005) on HCT-116 cells, respectively.

**Figure 3:**
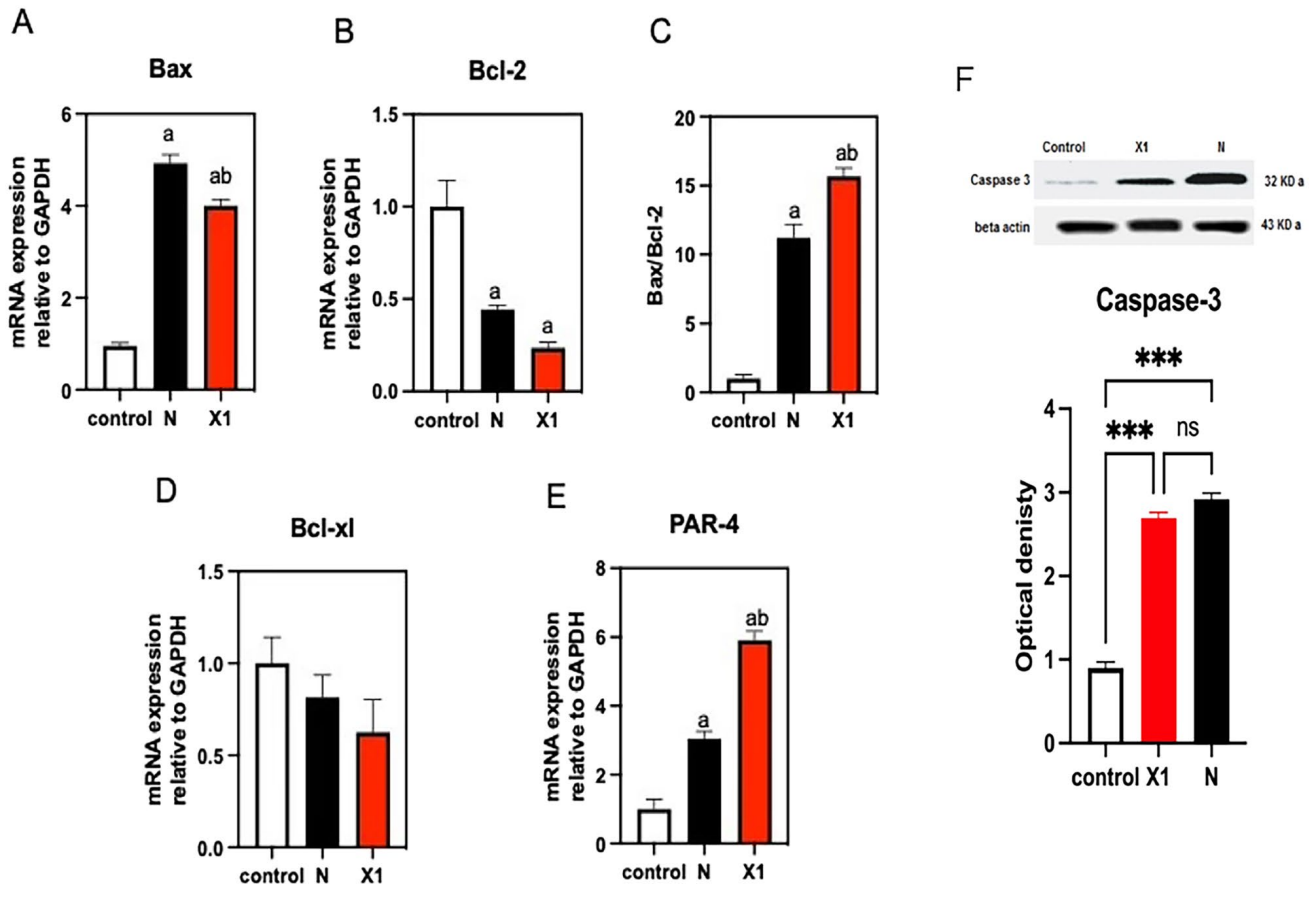
Effect of treatment with control, niclosamide, and X1 on mRNA expression of apoptotic genes and western blotting of Caspase-3 on HCT-116 cells. (**A**) BAX expression, (**B**) Bcl-2 expression ,(**C**) BAX/Bcl-2 ratio, (**D**) BCL-xl, (**E**) PAR-4, and (**F**) caspase-3. Results are expressed as the means ± SD of two independent experiments performed in duplicate. The statistical significance of the results was analyzed using an unpaired t-test for mRNA expression of apoptotic genes and a one-way ANOVA for wester blotting of caspase. (a) Significantly different from the control group; (b) Significantly different from the niclosamide-treated group at P ˂ 0.05.

Our results indicated that N and X1 modulate the BAX, Bcl-2, Bcl-xL, and PAR-4 gene expressions. As shown in Fig. 3, N and X1 significantly upregulated apoptotic genes and downregulated antiapoptotic gene expression relative to the control. By calculating and studying the apoptotic index (BAX/Bcl-2 ratio), compound N showed a tenfold increase relative to the control, and compound X1 showed significantly the highest BAX/Bcl-2 ratio in comparison to the power (15-fold increase).

#### Western blotting of caspase-3

Niclosamide and X1 on human colon cancer cells HCT-116 showed significantly higher caspase-3 protein levels than control, with no significant difference between caspase-3 levels in niclosamide and X1 (Fig. 3F).

#### Annexin V assay for the assessment of apoptosis

**Figure 4:**
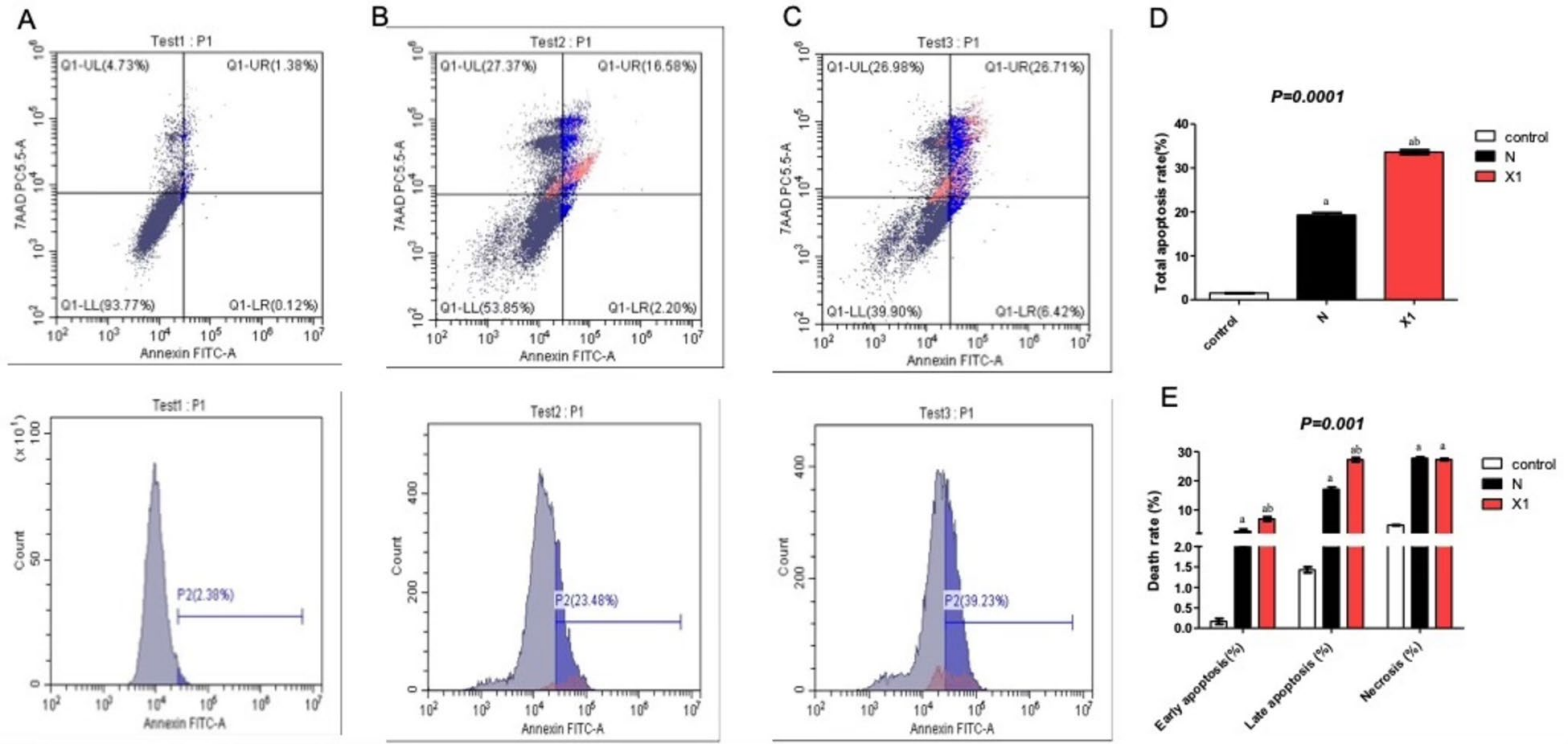
Effect of treatment with niclosamide and X1 on the total apoptotic ratio using flow cytometry in HCT 116 cells. Flow cytometry scatterplots for (**A**) control, (**B**) Niclosamide, (**C**) X1, (**D**) quantitative analysis of the total apoptosis rate, and (**E**) quantitative analysis of early, late apoptosis and Necrosis rates. Results are expressed as means ± SD of two independent experiments performed in duplicate. The statistical significance of the results was analyzed using a one-way ANOVA followed by Tukey’s multiple comparison test and a two-way ANOVA followed by Bonferroni test (a) significantly different from the control group, (b) significantly different from Niclosamide at P ˂ 0.05.

Further analysis of apoptosis by flow cytometry for compounds Niclosamide and X1 on human colon cancer cells HCT-116 showed the flow cytometry scatterplots for control (Fig. 4A), Niclosamide (Fig. 4B), and X1 (Fig. 4C), and showed that the total apoptosis rate (%) for control untreated cells, Niclosamide, and X1 were 1.5, 18.78, and 33.1%, respectively (Fig. 4D). X1 significantly increased late and early apoptosis rates relative to both control and Niclosamide (Fig. 4E). Both Niclosamide and X1 produced a significant increase in necrosis rate relative to control (*p* = 0.001).

#### Cell cycle assay by flow cytometry

**Figure 5:**
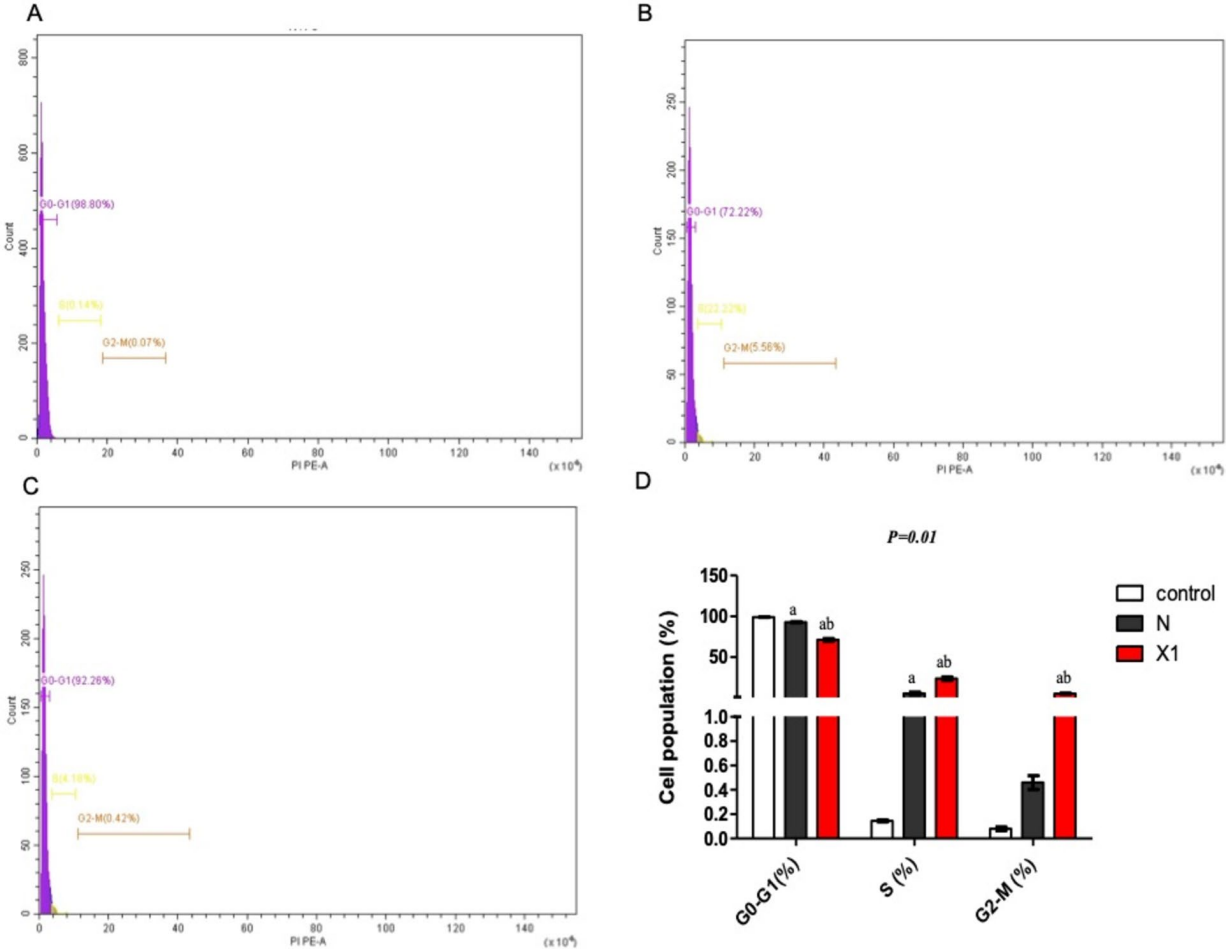
Cell cycle analysis of HCT-116 colon cancer cells (**A**) control, cells treated with (**B**) niclosamide, (**C**) X1, and (**D**) bar charts representing the percentage of cell population in the G0-G1, S, and G2-M phases of the cell cycle of untreated cells and after treatment with niclosamide and X1. Results are expressed as the means ± SD of two independent experiments performed in duplicate. The statistical significance of the results was analyzed using a two-way ANOVA followed by the Bonferroni test (a) significantly different from the control group, (b) significantly different from niclosamide at P ˂ 0.05.

Furthermore, Niclosamide and X1 were tested for cell cycle assays, and their effects on HCT-116 cells were analyzed using flow cytometry analysis. Figure 5 adopted the cell cycle analysis of control HCT-116 colon cancer cells (Fig. 5A), cells treated with niclosamide (Fig. 5B), and X1 (Fig. 5C). There was a significant decrease in G0/G1 with X1 relative to both control and Niclosamide (*p* = 0.01) and a significant increase in both the S phase and G2/M (Fig. 5D). Our data suggest that compound X1 exerts its antiproliferative activity by reducing the transition from G1 to the S phase as well as inhibiting the transition from G2 to the M phase also inducing apoptotic cell death.

### Molecular modeling study

#### In silico evaluation of physicochemical and ADME properties

An effective molecule needs to reach the target site in the body at a high enough concentration and remain there long enough to become bioactive in order for the anticipated biologic actions to take place. It is acknowledged that a medication's ideal properties should include both suitable pharmacokinetic properties and efficacy. The inherent pharmacological characteristics of a medicine as well as its absorption, distribution, metabolism, and excretion (ADME) aspects define its toxicity. High drug attrition rates in the later stages of pharmacological development have been associated with inadequate ADME features, such as the generation of hazardous metabolites.

Swiss *ADME* online was used to perform a computer evaluation of the synthesized compounds in order to assess their physicochemical and ADME properties.

Regarding Lipinski's rule for oral medications, a molecule with a molecular weight of less than 500 g/mol, a MlogP value of less than 5, and at least five H-donor and ten H-acceptor atoms has a better chance of being absorbed or penetrated. On the other hand, Veber's rule designates as drug-likeness limits polar surface area (PSA) 140 and rotatable bond count 10, respectively. It was noticed that all of the synthesized compounds have Lipinski zero violations, except molecular weight of X1 and X2 which exceed 500, in their physicochemical parameters and every chemical has rotatable bonds between 4 and 6 that indicate molecular flexibility for its bio target, (Table 1).

**Table 1:** Physicochemical properties based on Lipinski´s rule of five and number of rotatable bonds.

Cpd. no.	HBD	HBA	M logP	M.Wt	No. of Rot. bonds	Lipinski´s Violations	Veber’s violations
Xo	3	4	2.99	426.25	4	0	0
X1	3	7	2.59	573.45	6	1	0
X2	3	8	1.82	588.46	6	1	0
Niclosamide	2	4	2.44	327.12	4	0	0

Additionally, each hit passes muster according to the Veber screening specifications.

The topological polar surface area (TPSA) is widely recognized as a reliable indicator of drug penetration through the blood–brain barrier (TPSA less than 60 Å2) and intestinal medication absorption (TPSA less than 140 Å2). Since all compounds' computed TPSA values are within the range that permits them to cross cell membranes, they all adhere to Veber's criterion. Moreover, the following formula was used to calculate absorption (% ABS): % ABS = 109 − (0.345 × TPSA). Table 2 indicates that the calculated percentage ABS of all these hits ranged from 60.77 to 76.68%, indicating that these synthetic derivatives may have the necessary cell membrane permeability and bioavailability.

**Table 2:** The topological polar surface area (TPSA), and % ABS.

Compd. No.	TPSA	% ABS
Xo	90.79	77.68
X1	136.55	61.89
X2	139.79	60.77
Niclosamide	95.15	76.17

Drug availability in plasma is measured by bioavailability, which is considered to be the main factor controlling absorption. Table 3 reveals the intriguing fact that all of the synthesized molecules have high bioavailability ratings that are on par with Niclosamide. It is clear that Xo exhibited substantial gastrointestinal absorption even while X1 and X2 showed a small level of absorption. Furthermore, none of them were able to pass through the blood–brain barrier, guaranteeing that the central nervous system would not be negatively impacted by these systemically focused compounds. Compounds known as pan-assay interference compounds (PAINS) give false-positive findings in high-throughput screening quite frequently. Rather than directly affecting a single target, PAINS typically responds non-specifically to a wide range of biological targets. Examining any PAINS notifications pertaining to the recently created derivatives is crucial. After doing a PAIN, Swiss ADME discovered that there were no alarms on any of the hits. Normalised to range from 1 (very simple) to 10 (very difficult), the SwissADME Synthetic Accessibility (SA) Score is primarily based on the concept that the frequency of molecular fragments in "really" accessible compounds correlates with the ease of synthesis. Analogues' SA ratings ranged from 2.59 to 3.77, indicating the possibility of synthesizing these chemicals on a wide scale (Table 3). Based on the findings of the in silico ADME prediction study, the synthesized compounds exhibit the computational evaluation and are, thus, viewed as a pharmacologically active framework that should be taken into account when chasing potential hits.

**Table 3:** Pharmacokinetic properties and medicinal chemistry parameters.

Compd no.	GI Absorption	BBB Permeation	P-gp substrate	Bioavailability Score	PAINS Alerts	Synthetic Accessibility
Xo	high	No	No	0.55	0	2.97
X1	Low	No	No	0.55	0	3.77
X2	Low	No	No	0.55	0	2.59
Niclosamide	high	No	No	0.55	0	3.90

**Figure 6:**
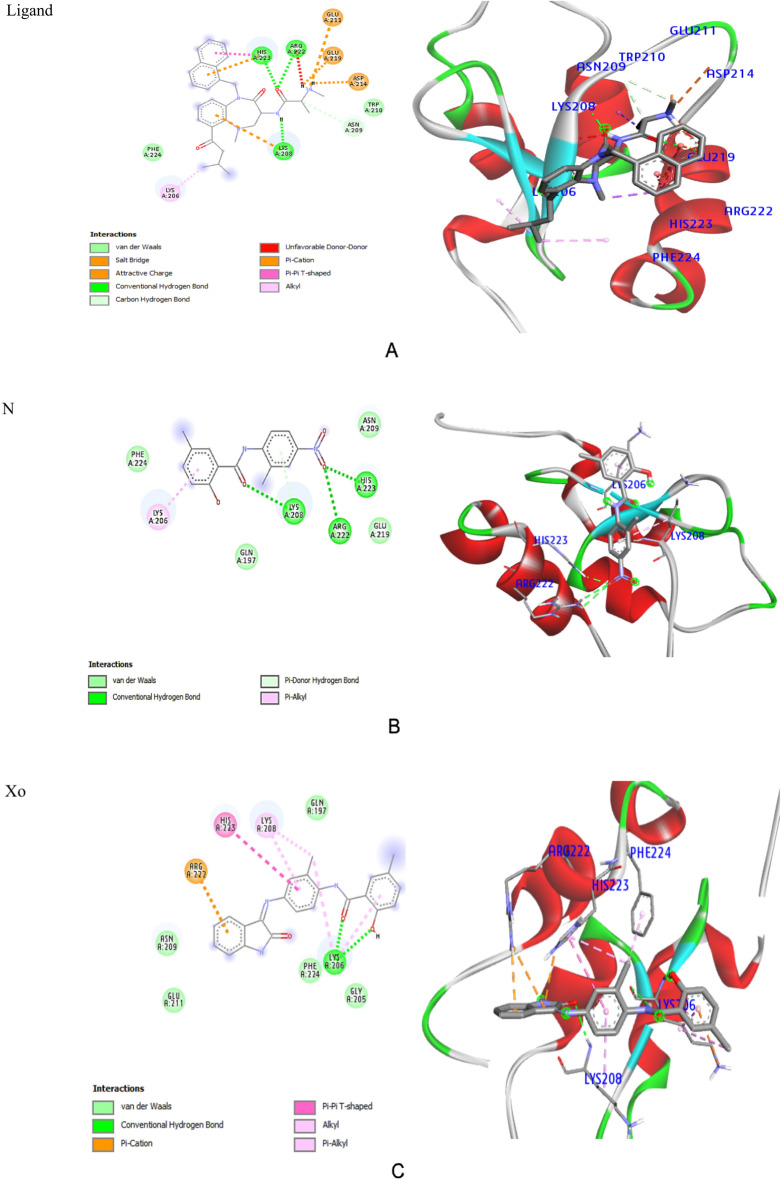

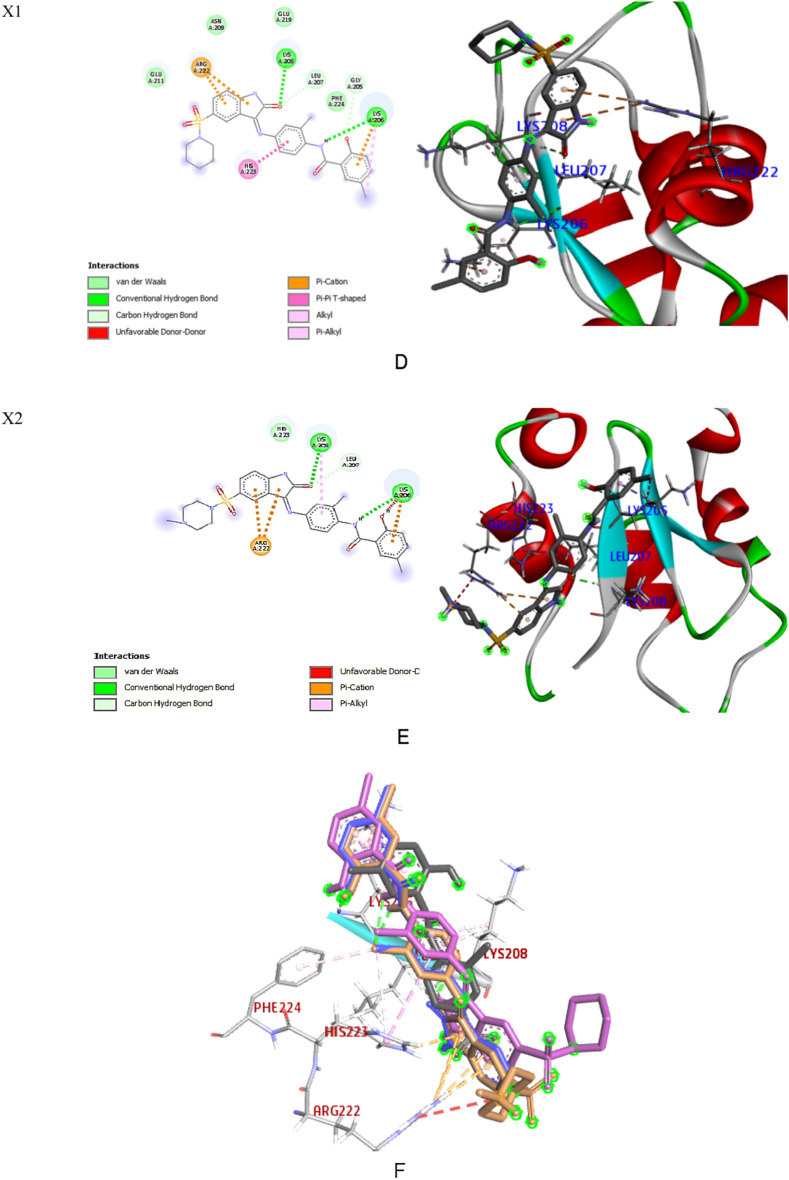
(**A**) 2D and 3D interactions of Ligand with 4kju. (**B**) 2D and 3D interactions of Niclosamide (N) with 4kju. (**C**) 2D and 3D interactions of Xo with 4kju. (**E**) 2D and 3D interactions of X1 with 4kju. (**E**) 2D and 3D interactions of X2 with 4kju. (**F**) Superimposition of NIC (black), Xo (blue), X1 (violet) and X2 (light brown) in the active site of XIAD BIR2 domain.

The pharmacological properties, brain penetration, and human intestinal absorption (HIA) of all the produced compounds and the standard drug (N) were displayed using the BOILED Egg diagram by the Swiss ADME online tool, as shown in Supplementary 5. The diagram's yellow (the yolk) section represents the region with the highest likelihood of entering the brain, while the white area represents the region with the best possibility of being absorbed by the human gastrointestinal tract. The whitish region included niclosamide and **Xo**, indicating gastrointestinal absorption. They are suitable candidates since they are not P-gp substrates. The research revealed that none of the tested substances, including niclosamide, are in the yolk region, indicating that they have not penetrated the *BBB* to reach the central nervous system and will not cause any adverse effects. Molecular docking of ligands with free binding energy: − 6.2 kcal/mol) reveals 3 H bonds with amide functionality: 2 HBA bind carbonyl-O with Arg222 and His223 and one HBD between NH and Lys208. Naphthyl moiety shared in binding to His223 by Pi-cation and Pi-Pi T-shaped interactions; phenyl moiety also shared in binding to Lys208 via Pi-cation. Moreover, the terminal amino group binds to both Asp214 and Glu219 through salt bridge interactions; furthermore, it binds through an attractive charge to Glu211. Moreover, the isopropyl moiety binds with Lys206 through an alkyl interaction, whilst the other side chain binds through a carbon-hydrogen bond to Asn209 (Fig. 6A).

Free binding energy was disclosed by niclosamide: Three HB interactions were seen at − 5.9 kcal/mol: two HBAs between the O of the NO2 functionality and His223, Arg222, and a further HB interaction between carbonyl-O and Lys208. Additionally, there were several hydrophobic contacts with Phe224, Gln197, Glu219, and Asn209, while Lys206 showed Pi-alkyl interactions with the backbone phenyl ring (Fig. 6B). Compound **Xo** revealed free binding energy: − 6.5 kcal/mol. It intermingled via OH and carbonyl group with Lys206 by two hydrogen bonds. Phenyl ring of isatin interacted with Arg222 through pi-cation. Many interactions were found through alkyl, pi-alkyl, and pi-pi T-shaped interactions with His223, Lys208, and the centroid of the phenyl rings. Further hydrophobic interactions with the following amino acids: Phe224, Gln197, Glu211, and Asn209, Gly205 (Fig. 6C).

The most potent caspase3 activator **X1** fitted well into the ligand’s site within the BIR2 domain, recording the most favored free binding energy: − 6.8 kcal/mol among the other derivatives. Compound X1 showed one hydrogen bond donor between the NH of amide and Lys206 and another HBA between isatin-O and Lys208. Terminal phenyl and isatin moieties shared interactions with Lys206 and Arg222 via 3-pi-cation interactions. Binding also includes two carbon-hydrogen bonds with Leu207 and Gly205. The central phenyl shared a pi-pi T-shaped interaction with His223 while the terminal phenyl displayed a pi-alkyl interaction with Lys206. Multiple Van der Waals interactions with Gly204, Phe224, Glu219, Asn209, and Glu211 were also observed (Fig. 6D).

Docking of compound **X2** showed free binding energy: − 6.5 kcal/mol. It revealed two H-bonds linking isatin-O and NH of amide with Lys208. Two pi-cations link the isatin scaffold to Arg222, and the third pi-cation binds the terminal phenyl to Lys206. On the other hand, the central phenyl is shared by binding to Lys208 through pi-alkyl interaction. Isatin-O showed more contribution with Leu207 via the carbon-hydrogen bond in addition to the van der Waals interaction with His223 (Fig. 6E). According to the aforementioned findings, the phenyl ring substitution at position 5 of isatin plays a significant role in the criteria for cytotoxicity and apoptosis induction. Through multiple hydrophobic interactions with its piperidine moiety, the addition of sulfonyl piperidine at position 5 of the isatin scaffold (**X1**) significantly improves the activity and affinity compared to that of **Xo**. In contrast, **X2** with a group that is more prone to forming H bond (-NCH_3_) showed lesser affinity to the active site than **X1**.

Overlay docking alignment was also carried out between Niclosamide (black), **Xo** (blue), **X1** (violet) and **X2** (light brown) in the active site of the XIAP BIR2 domain that selectively binds caspase 3/7 to verify our target chemicals 's mode of action as caspase 3/7 activators and apoptosis inducers (Fig. 6F). From the previous table of interactions and figures, it is obvious that compound no. **X1** has common interactions with most of the amino acid residues of caspase 3/7 enzyme that Niclosamide interacted with. Moreover, **X1** showed additional interactions with another residue in the enzyme, this emphasizes the results of **X1** in the biological assays.

## Discussion

These findings are consistent with some earlier research. Niclosamide inhibited Wnt/b-catenin pathway activation, decreased downstream b-catenin signaling, decreased S100A4 mRNA and protein expression, and had antiproliferative effects in colorectal cancer HCT-116 cells, human hepatic cancer cell lines (HepG2), and mouse liver metastasis^21–23^. Additionally, treatment with niclosamide resulted in overexpression of the tumor suppressor miR-200 family and inhibition of the Notch signaling pathway in colon cancer HCT-116 cell lines^24^.

Previous studies have shown that niclosamide enhanced the cytotoxic action against MCF-7 breast cancer, inhibited mTORC1 signaling indirectly, caused apoptosis in vitro and tumor growth in vivo, and prevented the growth of breast cancer spheroids^25–27^. Additionally, it was shown that niclosamide prevented non-breast cancer stem cells from developing into cancer stem cells^28^. In this process, the signal transduction pathway IL6-JAK1-STAT3 is inhibited. Our results indicated that niclosamide and compound X1 induced an apoptotic effect via a significant downregulation of anti-apoptotic Bcl-2, an insignificant decrease of Bcl-xL, and a significant upregulation of apoptotic genes PAR-4 and BAX on HCT-116 cells relative to the control. The current study found that compound X1 had a considerably greater BAX/Bcl-2 ratio than niclosamide by measuring and analyzing the apoptotic index (BAX/Bcl-2 ratio). This data suggests that compound X1 and niclosamide induce apoptosis in cancer cells.

These findings are supported by other studies, which demonstrated that STAT3 regulates the survival of cancer cells by triggering antiapoptotic genes such as Bcl-2 and Bcl-xL^29,30^. Niclosamide decreases STAT3 transcriptional activity by preventing STAT3's phosphorylation and nuclear translocation in different cancer cells^7,31^. You et al.^11^ demonstrated that niclosamide not only prevented acquired radioresistance in vitro and in vivo but also stopped the activation of the STAT3/Bcl-2/Bcl-xL pathway caused by ionizing radiation in both radiosensitive and radioresistant human lung cancer cells^11,32^.

Niclosamide boosted AMP-activated protein kinase (AMPK) activity and lipid oxidation in the mitochondria of mouse livers and human liver cancer cells. This, in turn, decreased oxidative phosphorylation and enhanced adenosine triphosphatase activity^27,33^. Niclosamide's ability to uncouple mitochondrial phosphorylation, damage mitochondria in tumor cells, trigger apoptosis, and suppress the activities of multiple oncogenic pathways, including and regulating different tumor signaling pathways like Wnt/b-catenin, mTORC1, JAK/STAT3, NF-kB, and Notch pathway, has been linked to its anticancer activity^8,12,21,27^. Based on all of this information, Niclosamide appears to be a promising anticancer drug.

Previous studies reported that niclosamide inhibits TNF-α-induced NF-κB-dependent activity, increases ROS levels in AML cells, and enhances the sensitivity of lung cancer cells to ROS^12,13^. Nitrosative stress-mediated apoptosis is a crucial apoptotic pathway, which occurs in response to both intrinsic and extrinsic apoptotic processes associated with the emergence of numerous illnesses^34^.

In the present study, niclosamide treatment significantly increased the GSH content and significantly reduced NOx and MDA levels in HCT-116 cells compared to untreated cells. On the other hand, Compound X1 shows no significant decrease in MDA level and a significant reduction in NOx level compared to both the control and niclosamide.

The extremely active metabolism and proliferation of cancer cells usually result in hypoxia and elevated ROS levels in the tumor micro-environment^12^. Because pro-tumorigenic signaling occurs near the site of ROS production, the use of antioxidants or inhibitors that target this source of ROS creation may prevent carcinogenesis^35^. ROS production in HCT-116 cells was found to be decreased after treatment with niclosamide and the compound X1. This may be related to the antioxidant properties of niclosamide or its hybrid compound X1. These findings support earlier findings^36–38^. Niclosamide has a dose-dependent protective effect against liver fibrosis model caused by bile duct ligation and methotrexate-induced liver damage by reducing oxidative stress markers^37,38^. Niclosamide shields the mitochondria by having the ability to decouple oxidative phosphorylation, which reduces ROS and oxidative stress. Mitochondrial uncoupling induced by niclosamide triggers a decrease in the proton gradient across the inner mitochondria membrane and increases fatty acid oxidation. Increased lipid oxidation reduces fat buildup in all cells, reduces ROS generation, and reduces oxidative stress^36–38^. The observed changes in cells treated with niclosamide may be responsible for their anti-cancer activities in specific cancer types.

Increasing or reducing intracellular ROS levels could mitigate the conflicting effect of ROS on the development of cancer^39^. An effective strategy for preventing the early stages of tumor manifestation is to lower the intracellular ROS concentration by blocking the ROS generation pathway. Since cancer cells are more susceptible to increased intracellular ROS than normal cells, increasing the amount of cellular ROS may be another strategy for killing only cancer cells^40^.

The present result showed that niclosamide induces caspase-dependent apoptosis and cell cycle arrest in the G1 phase, which is consistent with previous studies. In head and neck squamous cell carcinoma cell lines, Niclosamide treatment resulted in an increase in let-7d and a decrease in the cell cycle regulator CDC34 expression, ultimately resulting in G1 phase arrest^10,41^.

Moreover, previous studies revealed that niclosamide is a powerful mitochondrial uncoupler that inhibits imperative cellular pathways, triggers caspase-dependent apoptosis, arrests the G1 cell cycle, and reduces cellular migration in head and neck cancer^10^, adrenocortical carcinoma^42^, oral squamous cell carcinoma^41^, and oesophageal cancer cells^43^.

Niclosamide has been shown to cause mitochondrial damage, increase ROS, and induce apoptosis in acute myelogenous leukemia (AML) cells by raising cytochrome c levels^13,42^. The altered mitochondrial function seen in cancer cells might explain the low toxicity of niclosamide in normal cells. Cationic substances like niclosamide will preferentially concentrate in cancer cells because their mitochondria are hyperpolarized relative to normal cells. Moreover, the acidic environment created by the high glycolytic rate in tumor cells may enhance the availability and activity of the drug^42^.

Moreover, Park et al.^44^, categorized niclosamide as a potent inducer of mitochondrial fission, which led to mitochondrial fragmentation and promoted both apoptotic and autophagic cell death. In addition, it was reported that the inactivation of NF-κB by niclosamide caused mitochondrial damage and modulated ROS generation, leading to the apoptosis of acute myelogenous leukemia cells^13^. In uveal melanoma cells, the increase in ROS and inhibition of NF-κB are independent mechanisms of niclosamide^45^.

Finally, Yo et al.^45^ demonstrated that niclosamide could interfere with a variety of metabolic pathways that alter redox regulation and biogenesis in cells that are developing ovarian cancer. These disturbances result in the loss of tumor stemness, activation of the intrinsic mitochondrial apoptotic pathway, and suppression of growth.

## Conclusion

Here, novel Niclosamide-isatin hybrids were created and synthesized, exhibiting cytotoxicity against human HepG-2, HCT-116, and MCF-7 cell lines. The most effective analogue, X1, demonstrated high cytotoxicity and good selectivity towards HCT-116. In HCT-116 cells, Niclosamide and its hybrid X1 provoke cell apoptosis and modulate the BAX, Bcl-2, BCL-xl, and PAR-4 gene expressions. The mechanism of action may involve arresting the cell cycle progression at the G1 phase and inducing apoptosis via mitochondria-dependent pathways. The molecular docking revealed that hybrid X1 has the lowest free energy and the maximum affinity for the active site of the XIAP BIR2 domain, which binds caspase 3/7 preferentially. This indicates that our target chemical acts as an apoptosis inducer and caspase-3 activator. These findings suggested that chemical X1, which has an apoptotic-mediated antiproliferative activity, may hold promise for the creation of novel anticancer medications.

## Materials and methods

### Materials

All solvents were freshly distilled and purified according to standard procedures^46^. The used chemicals, 5-chlorosalicylic acid and 2-chloro-4-nitroaniline were received from Sigma Aldrich, while isatin from Panreac, EU. Also, the solvents such as methanol were obtained from El Salam Company, Egypt, Ethanol (International Company, Egypt), Acetic acid from El Nasr Company. Inaccurate melting points (M.Ps) of the novel designed compounds are determined and reported on the digital Gallen Kamp MFB-595 instrument. All reactions were routinely checked with thin-layer chromatography (TLC) of Merck Silica Gel 60 F254 (0.25 mm thick) and visualization with a UV lamp. A Shimadzu 440 spectrophotometer is used to measure the IR spectra at the range, 400–4000 cm^−1^. A Bruker (400 and 101 MHz) spectrometer is used to evaluate the ^1^H and ^13^C signals in the NMR spectra and are recorded relative to deuterated solvent signals only in dimethyl sulfoxide (DMSO-*d*_6_). Values of the chemical shift were listed as *δ* ppm units. Mass spectra were performed on a Shimadzu GS/MSQP 2010 plus spectrometer at 70 eV. The substituted indoline-2,3-dione, 5-(piperidin-1-ylsulfonyl)indoline-2,3-dione and 5-((4-methylpiperazin-1-yl)sulfonyl)indoline-2,3-dione were prepared according to the previous work^47–49^ and used as reagents. Also, Niclosamide was synthesized by the reaction of 5-chloros-2-hydroxybenzoic acid and 2-chloro-4-nitroaniline according to the reported method^50^.

### Synthesis of New Hybrids

#### Synthesis of *N*-(4-amino-2-chlorophenyl)-5-chloro-2-hydroxybenzamide (Niclosamide amine)

This intermediate was synthesized according to literature^51^, a solution of Niclosamide (2 g, 5 mmol) and Zn dust (0.46 g, 7.08 mmol) in MeOH (12.5 mL) were treated with AcOH (12.5 mL) slowly. A slight exotherm was noted during the early heating. Precipitate was observed in the reaction mixture, after 5 min, and Me OH (5 mL) was added every 30 min for 2 h to facilitate stirring. After 3 h, the reaction mixture was filtered through a plug of Celite to remove the zinc and concentrated to afford the desired solid powder. M.p. 220–222 °C; Pale yellow powder; Yield = 100%; IR: *υ*/cm^-1^: 3437, 3358 ((NH_2_, OH), 3283 (NH amide), 3040 (CH-aromatic), 1680 (C=O). Anal. Calc. for **C**_**13**_**H**_**10**_**Cl**_**2**_**N**_**2**_**O**_**2**_ (297.14): C, 52.55; H, 3.39; N, 9.43. Found: C, 52.53; H, 3.38; N, 9.45.

### Synthesis of Niclosamide-Isatin Hybrids (X_o_, X_1_, and X_2_)

#### General procedure

Niclosamide amine (0.3 g, 1 mmol) is added to an acetic acid-catalyzed solution of indoline-2,3-dione, 5-(piperidin-1-ylsulfonyl) indoline-2,3-dione, or 5-((4-methyl piperazine-1-yl)sulfonyl) indoline-2,3-dione in EtOH absolute. The resulting mixture was heated under reflux (monitored by TLC) for 3–6 h until the condensed reaction was completed. To acquire the desired Schiff's bases, the new precipitation was collected by filtering out and recrystallizing from EtOH / dioxane (1:2) to obtain the pure desired products.

#### 5-Chloro-*N*-(2-chloro-4-((2-oxoindolin-3-ylidene)amino)phenyl)-2-hydroxybenzamide (Xo)

M.p. 250–252 °C; Light orange powder; Yield = 84% (0.36 g); IR: *υ*/cm^-1^: 3220, 3182, 3159 (br. OH, NH-amide), 3086 (CH.aromatic), 1708 (C=O), 1631 (C=N). ^1^H NMR (DMSO-*d*_*6*_) *δ*/ppm: 6.56 (d, *J* = 8.0 Hz, 1H, Ar–H), 6.78 (t, *J* = 7.4 Hz, 1H, Ar–H), 6.89 (d, *J* = 8.0 Hz, 1H, Ar–H), 7.04 (s, 1H, Ar–H), 7.06 (s, 1H, OH; D_2_O exchangeable), 7.27 (d, *J* = 6.8 Hz, 1H, Ar–H), 7.36 (t, *J* = 7.8 Hz, 1H, Ar–H), 7.48 (d, *J* = 8.0 Hz, 2H, Ar–H), 7.97 (d, *J* = 6.8 Hz, 1H, Ar–H), 8.45 (s, 1H, Ar–H), 10.91, 11.02 (2s, 2H, 2NH; D_2_O exchangeable); ^13^C NMR (DMSO-*d*_*6*_) *δ*/ppm: 112.7, 113.3, 116.2, 117.8, 119.1, 119.9, 122.4, 123.3, 125.2, 125.9 (C–Cl), 129.4, 130.1, 132.6, 133.9, 135.3 (C–Cl), 138.9, 147.6, 151.2 (C–OH), 156.3 (C=N), 163.4 (C=O), 163.9 (C=O); MS m/z (%): 427 (M^+2^ , 18.68), 425 (M^+^, 39.48), 380 (100.00), 331 (89.10), 264 (48.02). Anal. Calc. for **C**_**21**_**H**_**13**_**Cl**_**2**_**N**_**3**_**O**_**3**_ (426.25): C, 59.17; H, 3.07; N, 9.86. Found: C, 59.83; H, 3.01; N, 9.37. Found: C, 59.43; H, 3.04; N, 9.47.

#### 5-Chloro-*N*-(2-chloro-4-((2-oxo-5-(piperidin-1-ylsulfonyl)indolin-3-ylidene)amino)phenyl)-2-hydroxybenzamide (X_1_)

M.p. 290–292 °C; Light red powder; Yield = 94% (0.53 g); IR: *υ*/cm^-1^: 3456, 3438, 3309 (br. OH, NH-amide), 2940, 2856 (CH. aliphatic), 1734 (C=O), 1616 (C=N). ^1^H NMR (DMSO-*d*_*6*_) *δ*/ppm: 1.32 (s, 2H, CH_2_-pipridinyl), 1.46 (br. s, 4H, 2CH_2_-pipridinyl), 2.65 (br. s, 4H, 2CH_2_-pipridinyl), 6.75 (s, 1H, Ar–H), 7.05 (s, 1H, OH; D_2_O exchangeable), 7.13 (dd, *J* = 8.0, 4.2 Hz, 1H, Ar–H), 7.35–7.48 (m, 2H, Ar–H), 7.64 (d, *J* = 5.6 Hz, 1H, Ar–H), 7.70 (d, *J* = 8.0 Hz, 1H, Ar–H), 7.86–7.87 (m, 1H, Ar–H), 7.97 (s, 1H, Ar–H), 8.49 (s, 1H, Ar–H), 11.36, 11.47 (2s, 2H, 2NH; D_2_O exchangeable); ^13^C NMR (DMSO-*d*_*6*_) *δ*/ppm: 23.4 (CH_2_-pipridinyl), 25.3 (2CH_2_-pipridinyl), 47.1 (2CH_2_-pipridinyl), 112.7, 112.9, 116.2, 118.5, 119.7, 124.0, 128.8, 129.0 (C–Cl), 130.4, 133.5, 134.4 (C–Cl), 137.0, 138.0, 147.2, 151.1, 155.4 (C–OH), 159.9 (C=N), 163.0 (C=O), 164.2 (C=O); MS m/z (%): 574 (M^+2^ , 8.14), 572 (M^+^, 3.27), 380 (100.00), 306 (82.33), 278 (19.54). Anal. Calc. for **C**_**26**_**H**_**22**_**Cl**_**2**_**N**_**4**_**O**_**5**_**S** (573.45): C, 54.46; H, 3.87; N, 9.77. Found: C, 54.13; H, 3.98; N, 9.98.

#### 5-Chloro-*N*-(2-chloro-4-((5-((4-methylpiperazin-1-yl)sulfonyl)-2-oxoindolin-3ylidene)amino) phenyl)-2-hydroxybenzamide (X_2_)

M.p. 300–302 °C; Deep red powder; Yield = 92% (0.54 g); IR: *υ*/cm^-1^: 3415, 3296 (br. OH, NH-amide), 3099 (CH.aromatic), 2942, 2855 (CH. aliphatic), 1741 (C=O), 1613 (C=N). ^1^H NMR (DMSO-*d*_*6*_) *δ*/ppm: 2.02 (s, 3H, N-CH_3_), 2.31–2.47 (m, 4H, 2CH_2_-piperazinyl), 3.05–3.09 (m, 4H, 2CH_2_-piperazinyl), 6.32–6-52 (m, 1H, Ar–H), 6.68 (d, *J* = 6.2 Hz, 1H, Ar–H), 7.01 (d, *J* = 6.4 Hz, 1H, Ar–H), 7.11 (s, 1H, OH; D_2_O exchangeable), 7.36 (s, 1H, Ar–H), 7.45 (d, *J* = 8.0 Hz, 1H, Ar–H), 7.64 (d, *J* = 8.0 Hz, 1H, Ar–H), 7.72 (s, 1H, Ar–H), 7.91 (d, *J* = 8.0 Hz, 1H, Ar–H), 7.97 (s, 1H, Ar–H), 10.37, 11.54 (2s, 2H, 2NH; D_2_O exchangeable); ^13^C NMR (DMSO-*d*_*6*_) *δ*/ppm: 44.1 (N-CH_3_), 47.1 (2 CH_2_-piperazinyl), 52.6 (2 CH_2_-piperazinyl), 113.3, 114.0, 117.1, 119.3, 119.6, 123.2, 123.6, 124.1, 126.7 (C–Cl), 127.2, 129.4, 130.1, 133.6 (C–Cl), 137.6, 139.6, 148.0, 154.6, 157.0 (C–OH), 159.9 (C=N), 164.3 (C=O), 165.8 (C=O). Anal. Calc. for **C**_**26**_**H**_**23**_**Cl**_**2**_**N**_**5**_**O**_**5**_**S** (588.46): C, 53.07; H, 3.94; N, 11.90. Found: C, 53.44; H, 3.84; N, 11.98.

### Biological assays in vitro studies

#### Human cancer cell line

Human Colon cancer cell line HCT-116, Human breast Cancer Cell line MCF-7 and Liver Cancer cell line HEPG-2, in addition to normal monkey kidney cell line Vero were obtained frozen in liquid nitrogen (− 180 °C) obtained from VACSERA (the Egyptian Company for Production of Vaccines, Sera and Drugs). The tumor cell line was maintained as monolayer cultures in DMEM supplemented with 10% FBS and 1% penicillin–streptomycin. The treatment protocol has been approved by the research ethics committee of the NCRRT, Cairo, Egypt (2A/22).

#### Cytotoxicity assay

The cytotoxicity of the novel hybrids was evaluated using sulforhodamine-B (SRB) method according to that of Skehan et al.^52^. In 96-well microtiter plates, cells were seeded at a concentration of 3 × 10^3^ cells/well. The cells were treated for 48 h with different concentrations (0, 6.25, 12.5, 25, and 50 μg/ml) of synthesized compounds and parent as control. The optical density (O.D.) of each well was measured spectrophotometrically at 570 nm using an ELISA microplate reader (TECAN Sunrise TM, Germany). The mean values estimated as the percentage of cell viability as follows:

O.D (treated cells)/O.D (control cells) × 100. The IC_50_ value (the concentration that produces 50% inhibition of cell growth) of each drug was calculated using dose–response curve-fitting models (Graph-Pad Prism software, version 5). The compound X1 was highly selective for the colon cancer cell line (HCT-116), so the colon cancer cell line was chosen for further investigations.

#### Preparation of cell-free media and cell lysate

Cells of the HCT-116 cell line were cultured in T75 flasks, left for 24 h, and then treated with IC_50_ concentration of the compounds Niclosamide as apparent and its analog X1 for 48 h. The medium was collected and used for the determination of NOx level. Cell pellets were prepared by removing the cells from the flasks by trypsinization and used for the determination of glutathione and MDA content. The treated and control cell pellets were collected, washed, and suspended in cold lysis buffer, then sonicated and centrifuged, and the clear supernatant was taken into another Eppendorf.

#### Determination of protein concentration

The Bradford method^53^ was used to determine the protein concentration in the medium and cell lysate. The procedure was based on the Coomassie brilliant blue G-250 dye creating a compound with the protein and being detectable spectrophotometrically at 595 nm. The concentration was then calculated using a standard calibration curve.

#### Determination of non-protein reduced thiols content (glutathione content)

Reduced glutathione (GSH) in cell lysate was determined according to the method of Ellman^54^, it is based on the reduction of Ellman's reagent [5,5′-dithio-bis-(2-nitrobenzoic acid)] by SH groups to form 1 mol of 2-nitro-5-mercaptobenzoic acid per mole of SH. The optical density was measured at 412 nm against a reagent blank and the results were expressed as μmol/mg protein.

#### Determination of total nitrate/nitrite (NOx)

Estimation of the total nitrate/nitrite (NOx) in cell culture media as a stable end product, nitrite was evaluated following Miranda et al. method^55^. The nitrate reduction by vanadium trichloride and detection by the acidic Griess reaction are the fundamentals of the test. A brightly colored product was produced after the diazotization of sulfanilic acid with nitrite at an acidic pH and coupling with N-(10-naphthyl) ethylenediamine and measured spectrophotometrically at 540 nm and expressed as nmol/mg protein.

#### Determination of lipid peroxidation

Lipid peroxidation products were determined by measuring malondialdehyde (MDA) levels in cell lysate using the method of Buege and Aust^56^. The principle mainly depends on the formation of thiobarbituric acid reactive substances, which have a pink color with absorption in spectrophotometry at 535 nm wavelength. The results were expressed as nmol/ mg protein.

#### Determination of mRNA expression of apoptotic genes

Using quantitative real-time PCR, the expression of BAX, Bcl-2, PAR-4, and BCL-xl genes (Table 4) in cells was quantified. The quality and amount of the total RNA were assessed using nanodrop (Thermo Fisher, UK) after it had been extracted from the control and treated cells with Trizol Reagent (Invitrogen, Carlsbad, CA). cDNA Reverse Transcription Kit (Applied Biosystems, Waltham, MA) was used to reverse-transcribe single-stranded RNA into complementary DNA. Using a thermocycler (Biometra, Germany), thermal cycling was started under the following conditions: 25 °C for 10 min, 37 °C for 120 min, 85 °C for 5 min, and 4 °C for ∞. Real-time PCR analysis was conducted using the thermo-cycler Step OneTM (Applied Biosystems). Each RT-reaction served as a template in a 20 μL PCR reaction containing 0.2 μmol/L of each primer and SYBR green master mix (Thermo Fisher Scientific, UK). Real-time PCR reactions were performed at 50 °C for 2 min, 95 °C for 10 min, followed by 45 cycles at 95 °C for 15 min and 56 °C for 1 min. The mRNA levels of these genes were normalized to GAPDH (ΔCT). The ΔCT was calibrated against an average of the control samples.

**Table 4:** Oligonucleotides used in the qPCR analysis.

Gene	Forward primer	Reverse primer
GAPDH	GTGGAGTCCACTGGCGTCTT	GCAAATGAGCCCAGCCTTC
BAX	CCT TTT CTA CTT TGC CAG CAAAC	GAG GCC GTC CCA ACCAC
Bcl-2	ATGTGTGTGGAGAGCGTCAACC	GCATCCCAGCCTCCGTTATC
BCL-xl	TCTGGTCCCTTGCAGCTAGT	TCCTTTCTGGGGAAGAGGTT
PAR-4	GCCGCAGAGTGCTTAGATGAG	GCAGATAGGAACTGCCTGGATC

#### Western blotting of Caspase-3

The ReadyPrep TM protein extraction kit (total protein) provided by Bio-Rad Inc (Catalog #163-2086) was employed according to manufacturer instructions and was added to each sample of the homogenized tissues of all different groups. Bradford Protein Assay Kit (SK3041) for quantitative protein analysis was provided by Bio Basic Inc (Markham Ontario L3R 8T4 Canada). A Bradford assay was performed according to manufacturer instructions to determine protein concentration in each sample. 20 μg protein concentration of each sample was then loaded with an equal volume of 2 × Laemmli sample buffer containing 4% SDS, 10% 2-mercaptoethanol, 20% glycerol, 0.004% bromophenol blue, and 0.125 M Tris HCl. The pH was checked and brought to 6.8. Each previous mixture was boiled at 95 °C for 5 min to ensure denaturation of protein before loading on polyacrylamide gel electrophoresis. Polyacrylamide gels were performed using TGX Stain-Free™ FastCast™ Acrylamide Kit (SDS-PAGE), which was provided by Bio-Rad Laboratories Inc Cat # 161-0181. The SDS-PAGE TGX Stain-Free FastCast was prepared according to manufacturer instructions. The gel was assembled in a transfer sandwich as follows from below to above (filter paper, PVDF membrane, gel, and filter paper). The sandwich was placed in the transfer tank with 1 × transfer buffer, which is composed of 25 mM Tris, 190 mM glycine, and 20% methanol. Then, the blot was run for 7 min at 25 V to allow protein bands to transfer from the gel to the membrane using BioRad Trans-Blot Turbo. The membrane was blocked in tris-buffered saline with Tween 20 (TBST) buffer and 3% bovine serum albumin (BSA) at room temperature for 1 h. The components of the blocking buffer were as follows; 20 mM Tris pH 7.5, 150 mM NaCl, 0.1% Tween 20, and 3% bovine serum albumin (BSA). Primary antibodies of caspase-3 (sc-56053, Santa Cruz Biotechnology, USA) were purchased. Primary antibodies were diluted in TBST according to manufactured instructions. Incubation was done overnight in each primary antibody solution, against the blotted target protein, at 4 °C. The blot was rinsed 3–5 times for 5 min with TBST. Incubation was done in the HRP-conjugated secondary antibody (Goat anti-rabbit IgG-HRP-1mg Goat mab-Novus Biologicals) solution against the blotted target protein for 1 h at room temperature. The blot was rinsed 3–5 times for 5 min with TBST.

The chemiluminescent substrate (Clarity TM Western ECL substrate Bio-Rad cat#170-5060) was applied to the blot according to the manufacturer’s recommendation. Briefly, equal volumes were added from solution A (Clarity western luminal/enhancer solution) and solution B (peroxidase solution). The chemiluminescent signals were captured using a CCD camera-based imager. Image analysis software was used to read the band intensity of the target proteins against control sample beta-actin (housekeeping protein) by protein normalization on the ChemiDoc MP imager.

#### Annexin V assay for the assessment of apoptosis

HCT-116 cells were plated (1 × 10^6^ cells/well) in six-well plates, allowed to attach overnight, and treated with the IC_50_ of the parent and synthesized analog for 24 h of treatment. The adherent and floating cells were collected, washed twice with (4 °C) PBS, and resuspended in 400 μL binding buffer. Annexin V-FITC apoptosis detection kit (Beckman Coulter, Brea, CA) was used as the manufacturer’s recommendation using a Beckman Coulter Epics XL Flow Cytometer.

#### Cell cycle assay by flow cytometry

Cell cycle DNA index kit (Beckman Coulter, California, USA) was used as the manufacturer’s recommendation using a Beckman Coulter Epics XL Flow Cytometer.

#### Docking studies

The crystal structure of the XIAP BIR2 domain complexes with a benzodiazepinone-based inhibitor (ligand) was retrieved from the protein data bank (PDB ID: 4KJU). Auto-Dock (MGL-tools) was used to determine the grid box dimension of targeted proteins. The grid box was exported in text format. In the meanwhile, the target enzyme was exported in PDQT format.

### Statistical analysis

Results were expressed as mean ± SD, Data were analyzed by one-way analysis of variance ANOVA followed by Tukey’s multiple comparisons tests using software Prism 5.0 (Graph Pad, San Diego, CA, USA) and two-way ANOVA followed by Bonferroni test (a) Significantly different from the control group, (b) significantly different from Niclosamide at P˂0.05.

### Supplementary Information

Supplementary Information 1.

Supplementary Information 2.

Supplementary Information 3.

Supplementary Information 4.

Supplementary Information 5.

Supplementary Information 6.

Supplementary Information 7.

## Data Availability

All data generated or analysed during this study is included in this published article and its supplementary information files.
