# Supplementary material for: Pharmacological investigation of new niclosamide-based isatin hybrids as antiproliferative, antioxidant, and apoptosis inducers
Source: Sci Rep. 2024 Aug 27;14:19818. doi: 10.1038/s41598-024-69250-5 (PMC11349906; doi:10.1038/s41598-024-69250-5)
Supplement: Supplementary file 2 — Supplementary Information 2. [file 41598_2024_69250_MOESM2_ESM.docx]

|   **Supplementary S2:** Synthetic pathway of the starting material niclosamide amine |
| --- |
|  |
