# Supplementary material for: Pharmacological investigation of new niclosamide-based isatin hybrids as antiproliferative, antioxidant, and apoptosis inducers
Source: Sci Rep. 2024 Aug 27;14:19818. doi: 10.1038/s41598-024-69250-5 (PMC11349906; doi:10.1038/s41598-024-69250-5)
Supplement: Supplementary file 3 — Supplementary Information 3. [file 41598_2024_69250_MOESM3_ESM.docx]

|  |
| --- |
|   **Supplementary S3:** Synthesis of the target bioactive material **X_O_**, **X_1_**, and **X_2_** |
