# Supplementary figures and images for: Pharmacological investigation of new niclosamide-based isatin hybrids as antiproliferative, antioxidant, and apoptosis inducers
Source: Sci Rep. 2024 Aug 27;14:19818. doi: 10.1038/s41598-024-69250-5 (PMC11349906; doi:10.1038/s41598-024-69250-5)

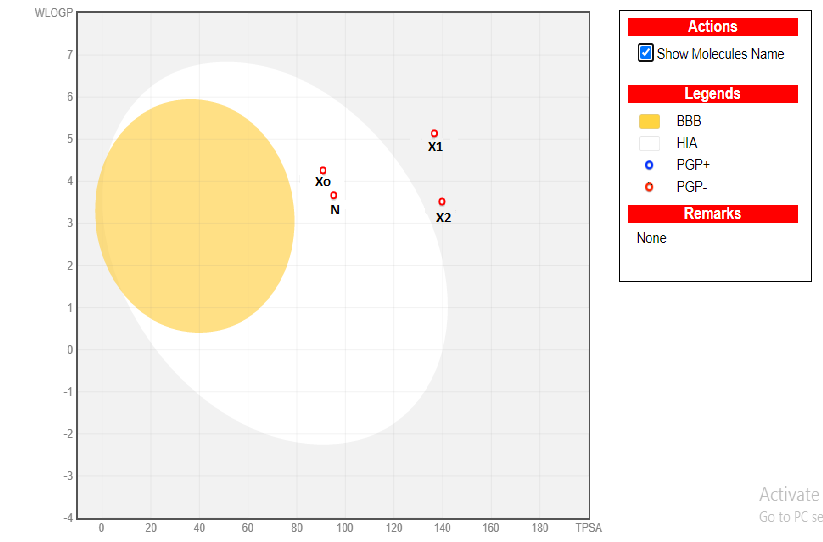


**Supplementary S5:** a BOILED-Egg diagram for niclosamide (N) and new niclosamide hybrids (Xo, X1 and X2)

Supplement: Supplementary file 5 — Supplementary Information 5. [file 41598_2024_69250_MOESM5_ESM.docx]

**
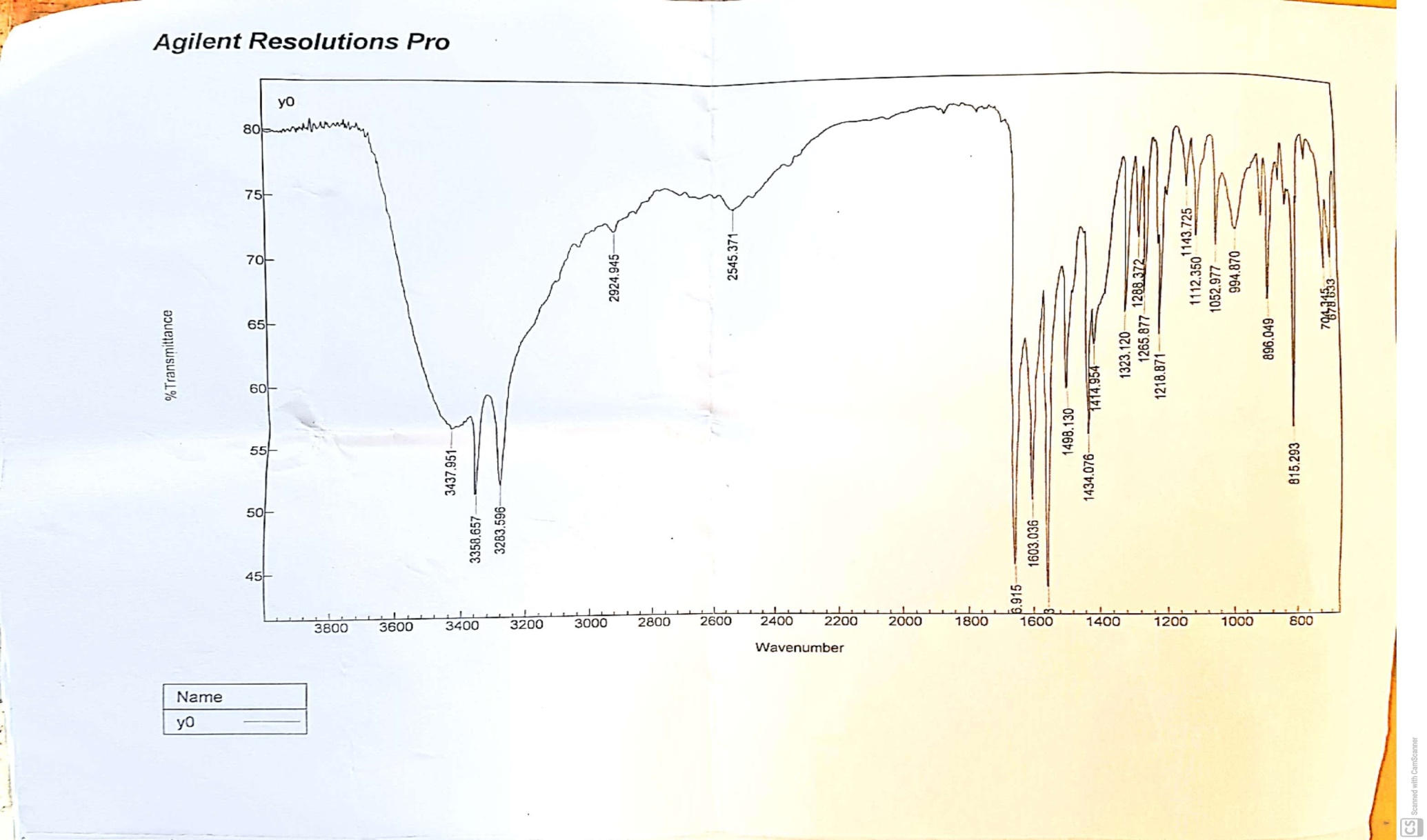
**


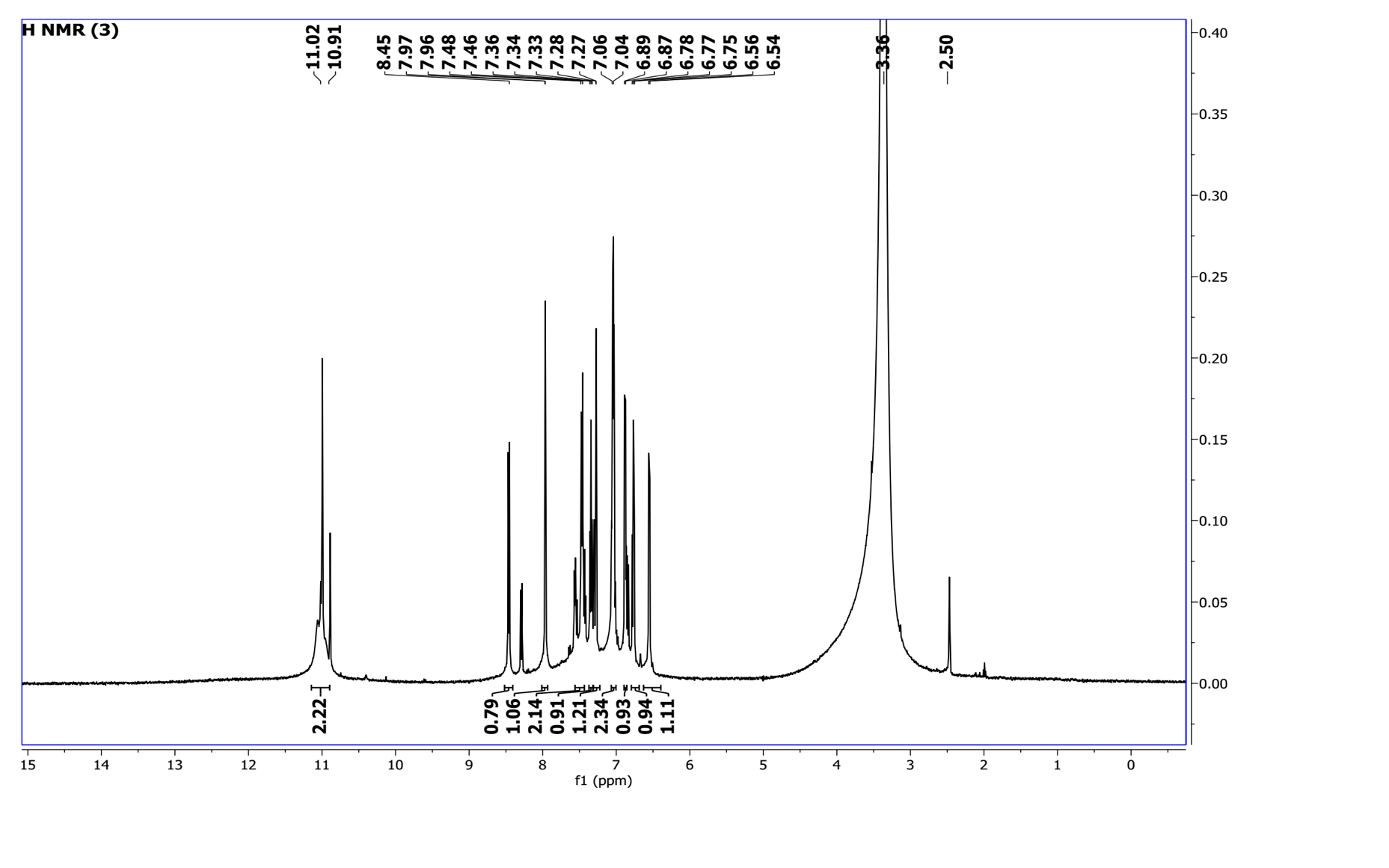


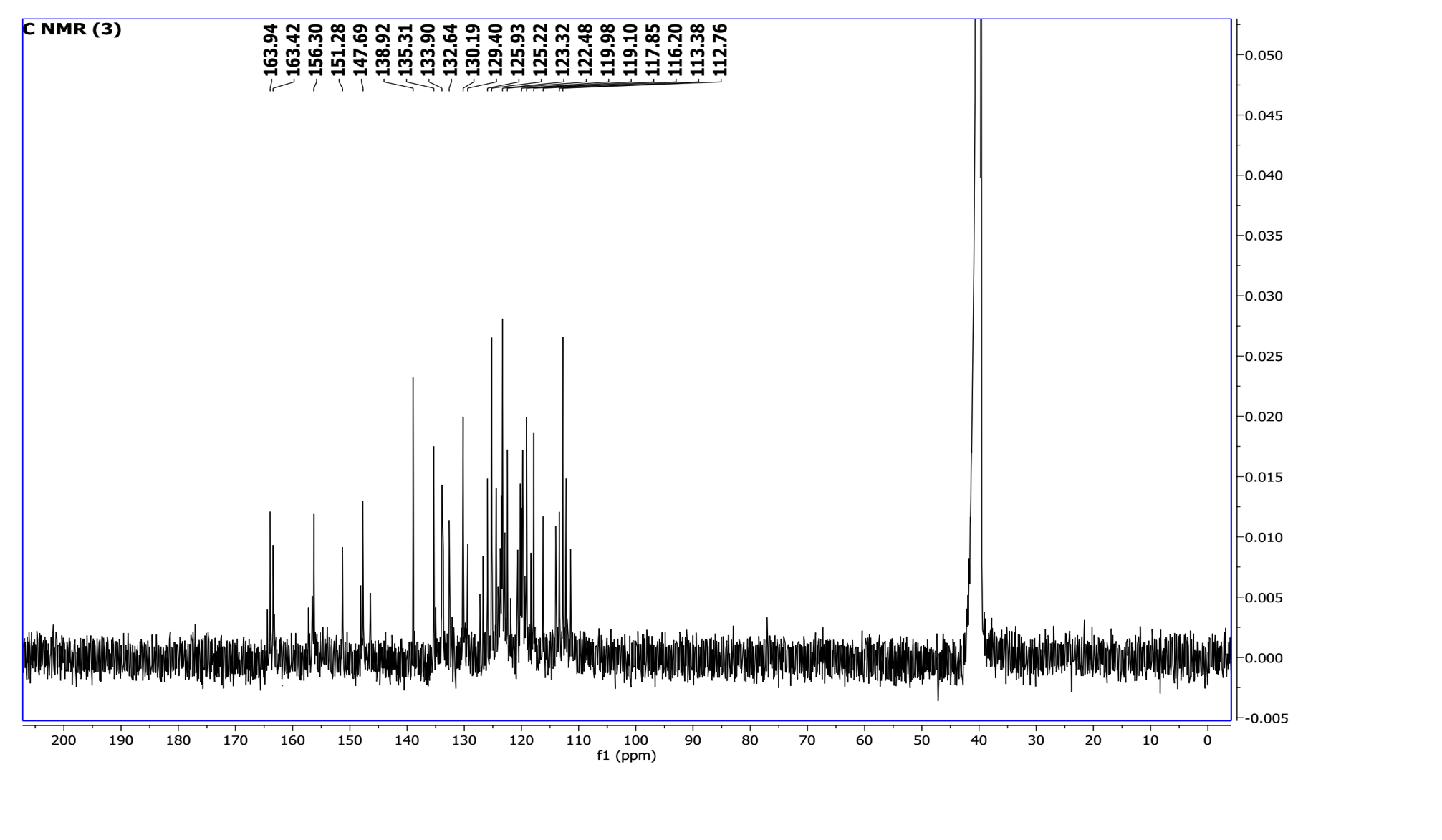


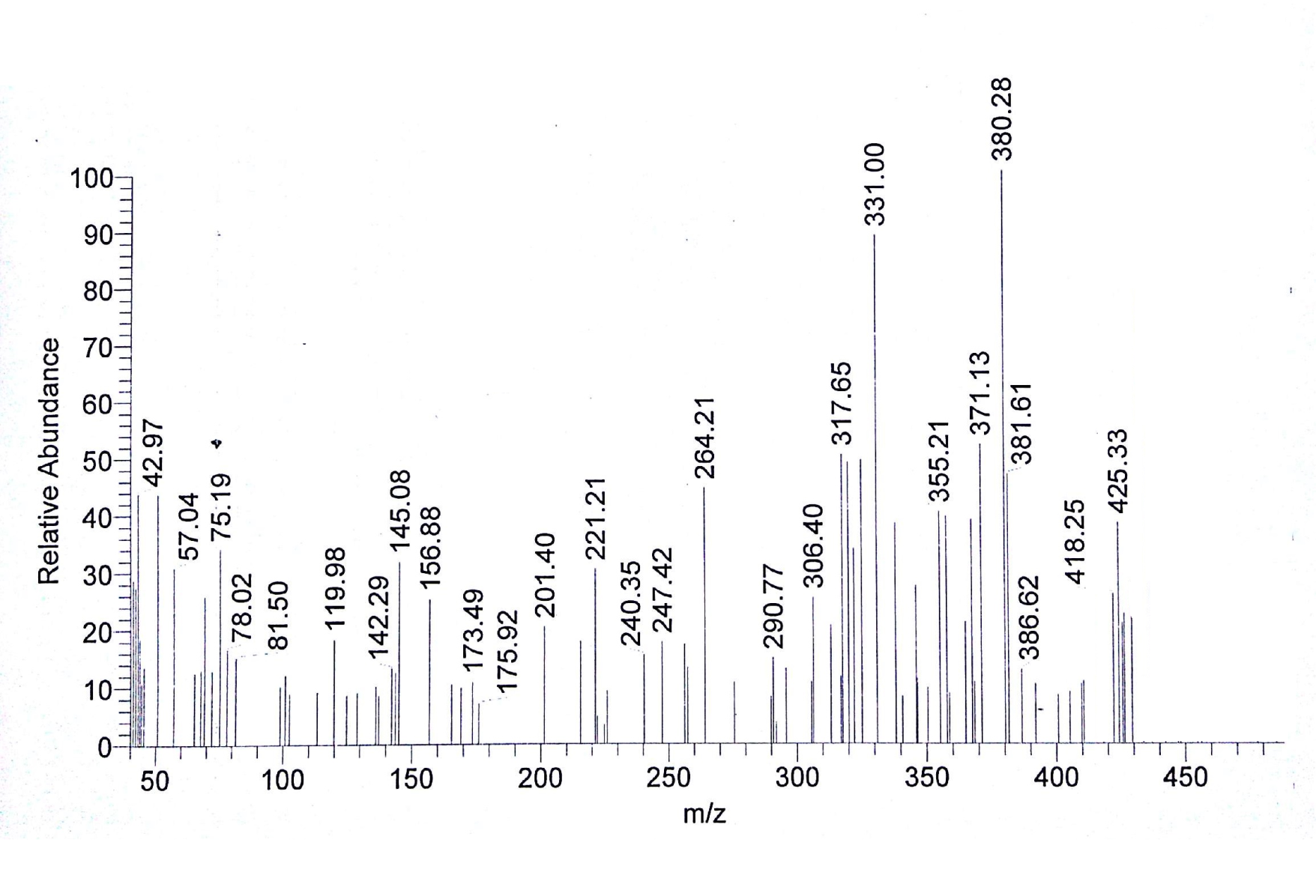


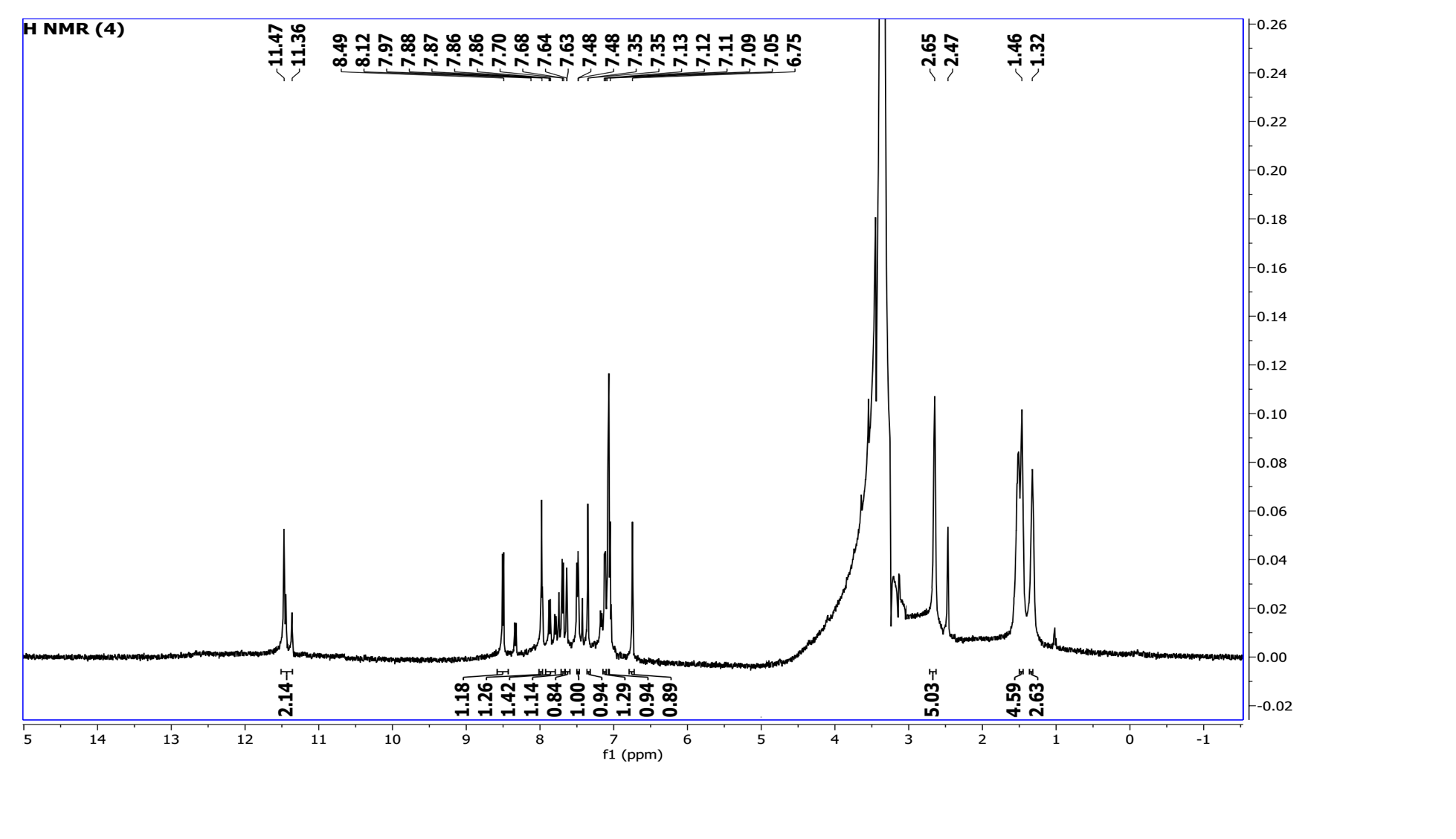


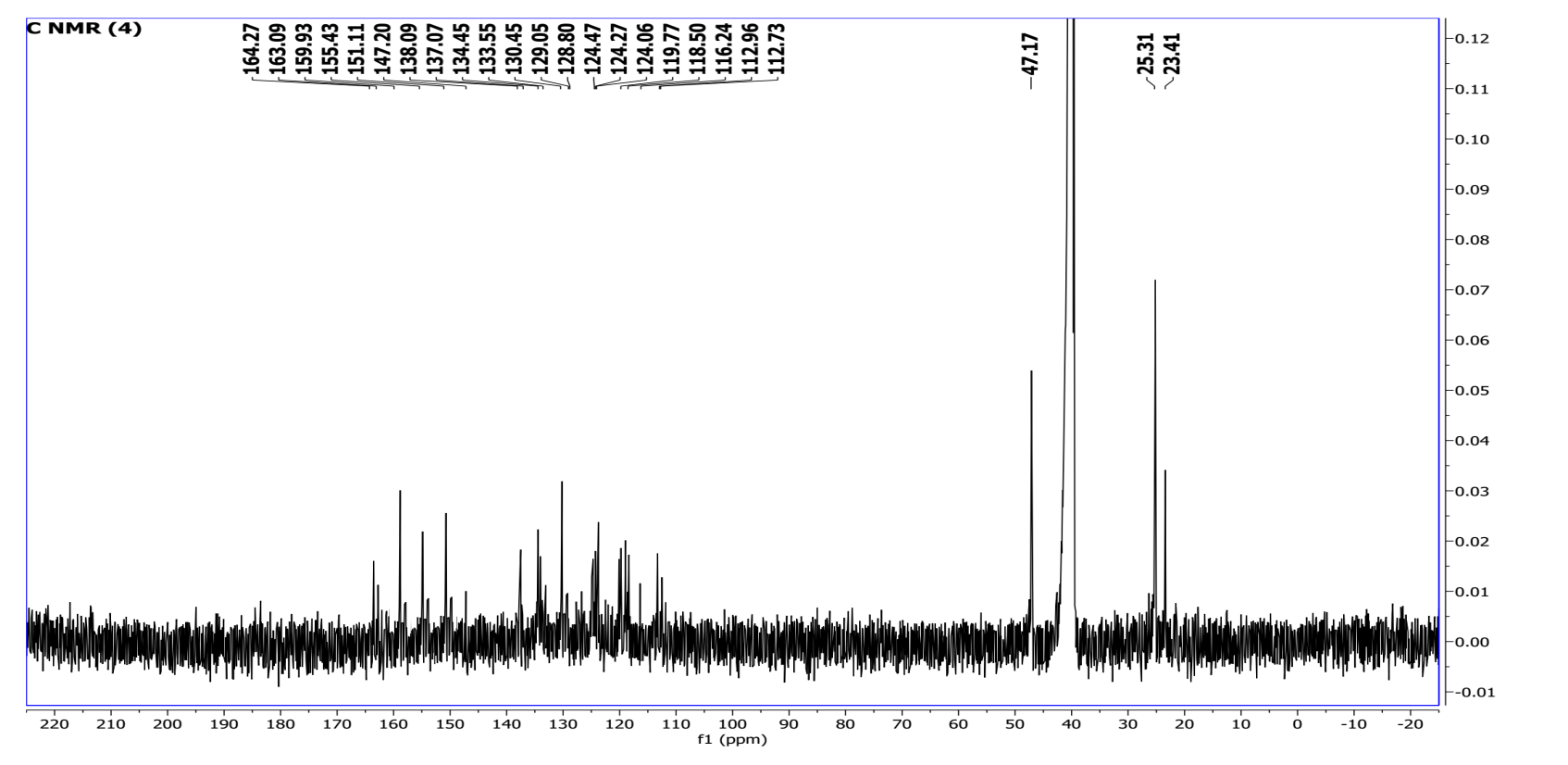


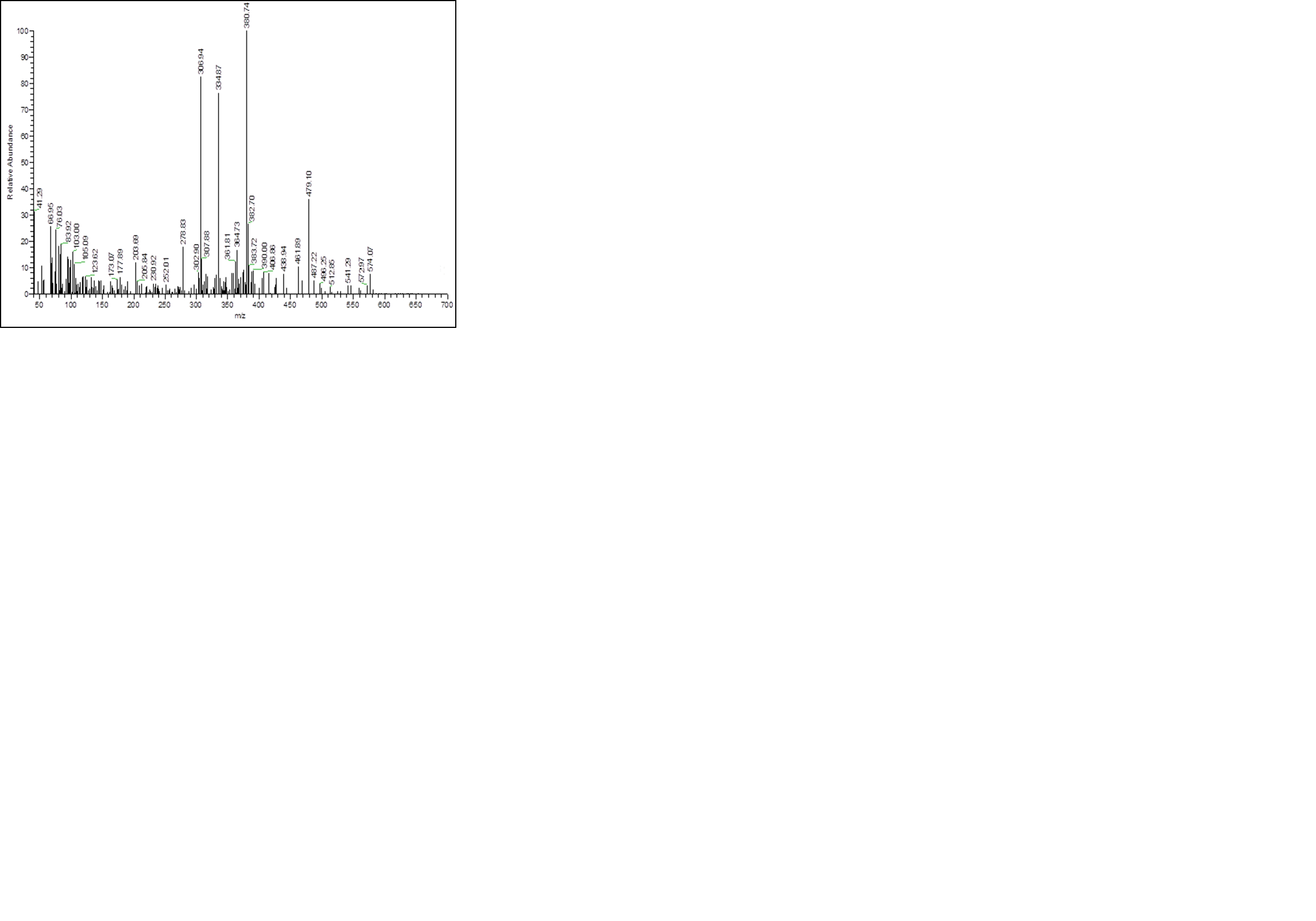


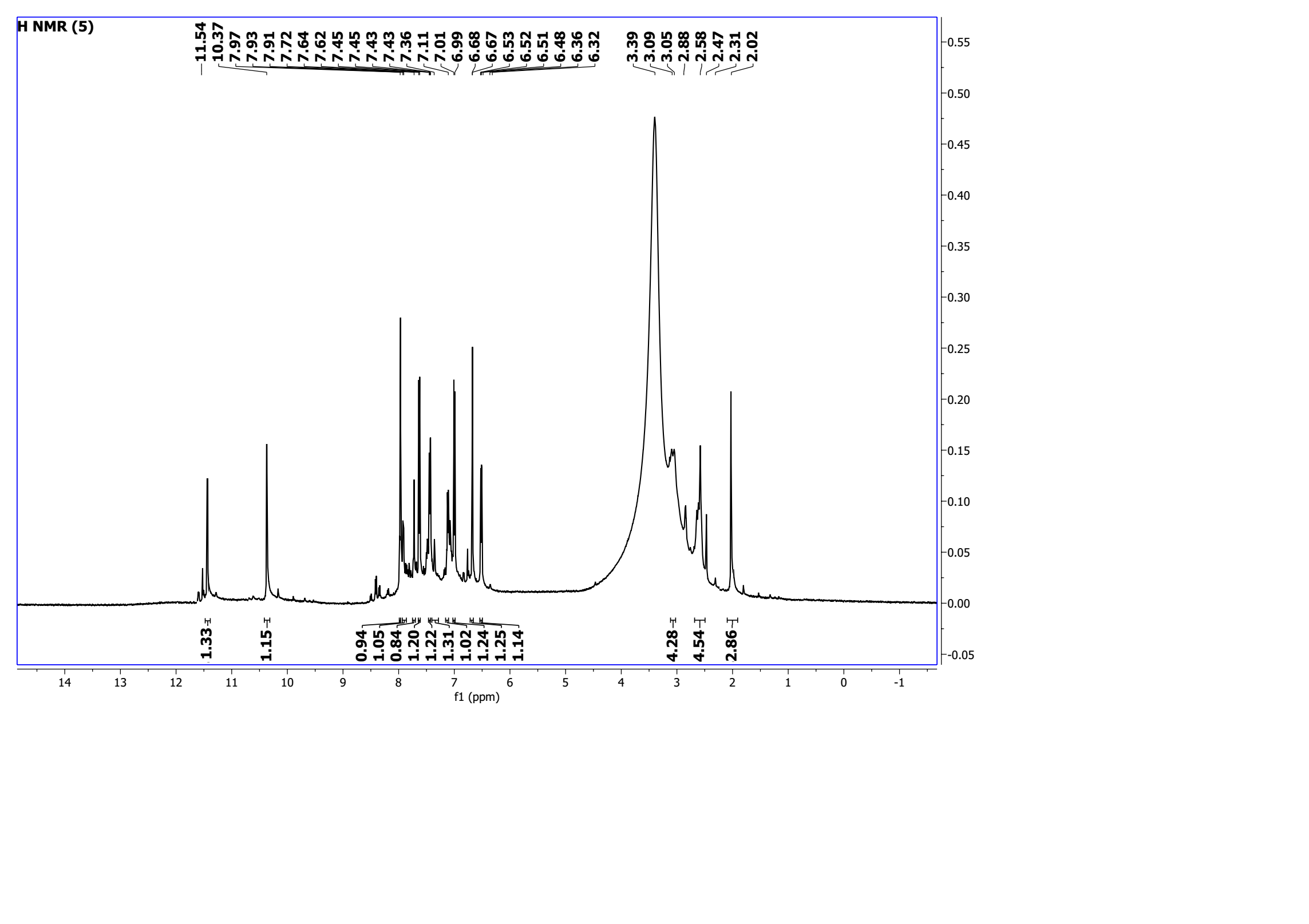


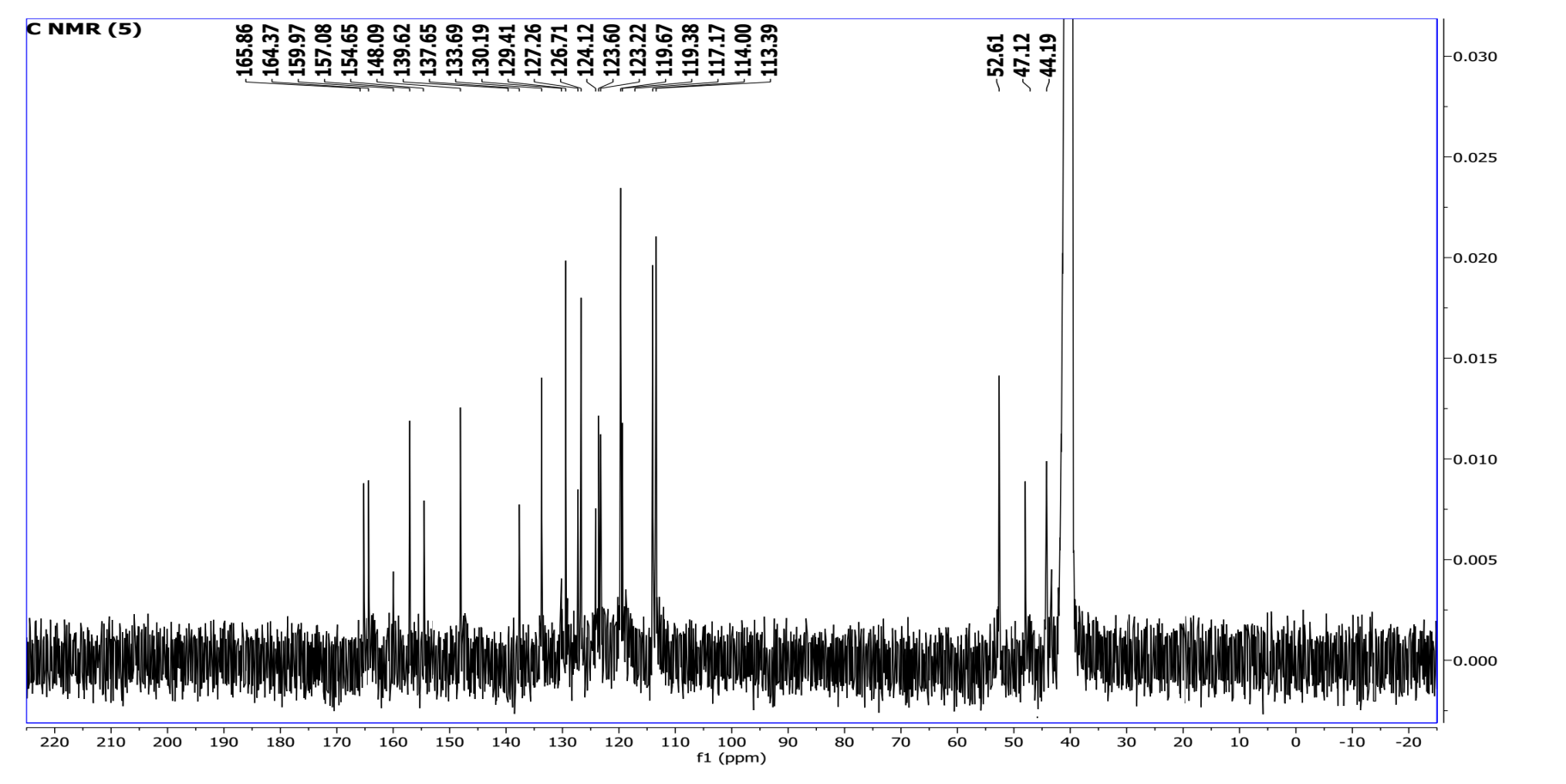

Supplement: Supplementary file 7 — Supplementary Information 7. [file 41598_2024_69250_MOESM7_ESM.docx]
