# Supplementary material for: Pharmacological investigation of new niclosamide-based isatin hybrids as antiproliferative, antioxidant, and apoptosis inducers
Source: Sci Rep. 2024 Aug 27;14:19818. doi: 10.1038/s41598-024-69250-5 (PMC11349906; doi:10.1038/s41598-024-69250-5)
Supplement: Supplementary file 6 — Supplementary Information 6. [file 41598_2024_69250_MOESM6_ESM.docx]

| **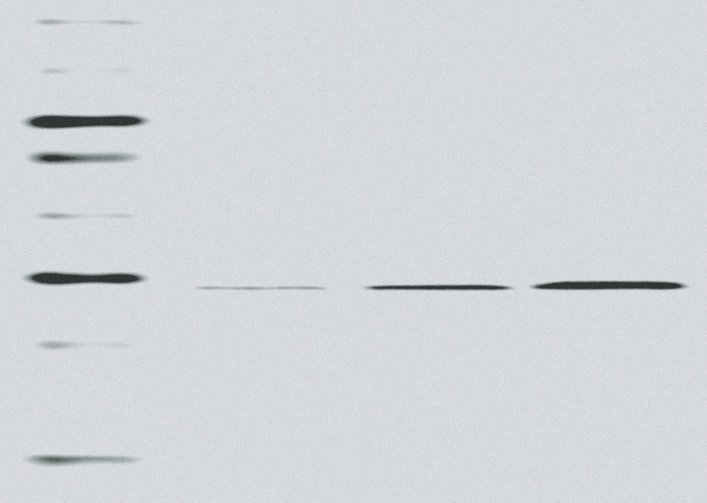**  caspase | 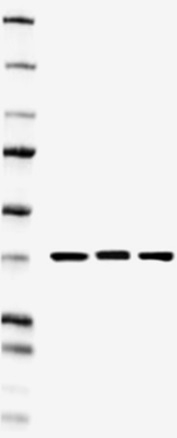  β-actin |
| --- | --- |

S6: Effect of treatment of control, Niclosamide, and X1 on mRNA expression of caspase-3
