# Supplementary material for: Pharmacological investigation of new niclosamide-based isatin hybrids as antiproliferative, antioxidant, and apoptosis inducers
Source: Sci Rep. 2024 Aug 27;14:19818. doi: 10.1038/s41598-024-69250-5 (PMC11349906; doi:10.1038/s41598-024-69250-5)
Supplement: Supplementary file 6 [file 41598_2024_69250_MOESM1_ESM.docx]

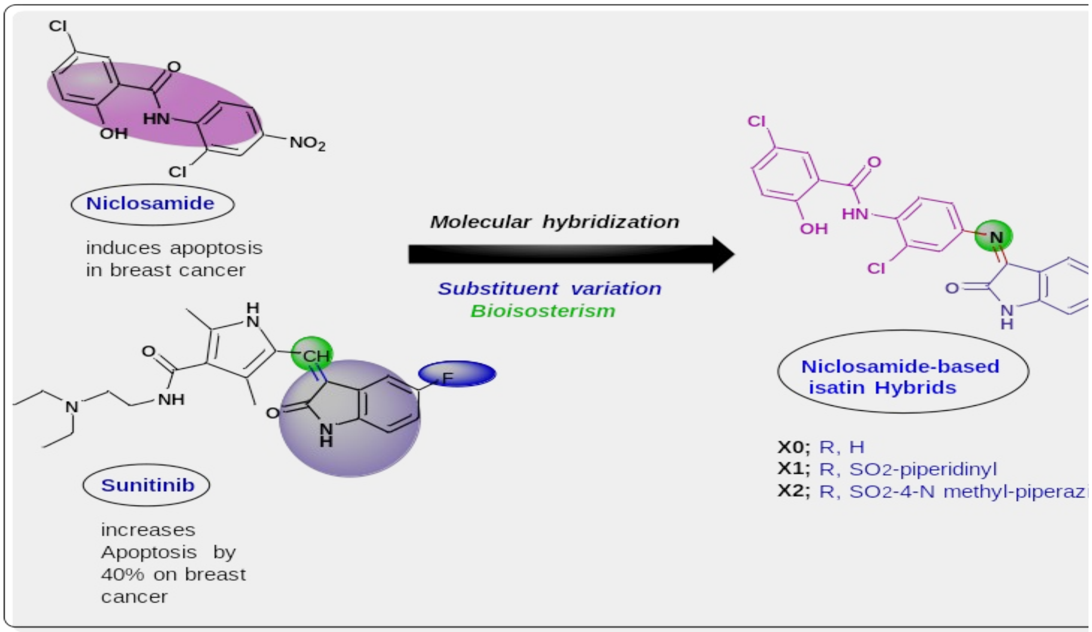

Supplementary S1: Rational of Niclosamide-isatin hybrids
